# HIV-1 latency reversal agent boosting is not limited by opioid use

**DOI:** 10.1172/jci.insight.185480

**Published:** 2024-11-22

**Authors:** Tyler Lilie, Jennifer Bouzy, Archana Asundi, Jessica Taylor, Samantha Roche, Alex Olson, Kendyll Coxen, Heather Corry, Hannah Jordan, Kiera Clayton, Nina Lin, Athe Tsibris

**Affiliations:** 1 Brigham and Women’s Hospital, Harvard Medical School, Boston, Massachusetts, USA.; 2 Department of Medicine, Boston University School of Medicine, Boston, Massachusetts, USA.; 3 Grayken Center for Addiction, Boston Medical Center, Boston, Massachusetts, USA.; 4 Department of Pathology, University of Massachusetts T.H. Chan School of Medicine, Worcester, Massachusetts, USA.; 5 Harvard Medical School, Boston, Massachusetts, USA.

**Keywords:** AIDS/HIV, Immunology, Addiction, T cells, Transcription

## Abstract

Opioid use may affect the HIV-1 reservoir and its reversal from latency. We studied 47 virally suppressed people with HIV (PWH) and observed that lower concentration of HIV-1 latency reversal agents (LRAs), used with small molecules that did not reverse latency, synergistically increased the magnitude of HIV-1 reactivation ex vivo, regardless of opioid use. This LRA boosting, which combined a second mitochondria-derived activator of caspases mimetic or low-dose PKC agonist with histone deacetylase inhibitors, generated more unspliced HIV-1 transcription than PMA with ionomycin (PMAi), the maximal known HIV-1 reactivator. LRA boosting associated with greater histone acetylation, modulated surface activation-induced markers, and altered T cell production of TNF-α, IL-2, and IFN-γ. HIV-1 reservoirs in PWH contained unspliced and polyadenylated virus mRNA, the ratios of which were greater in resting than total CD4^+^ T cells and corrected to 1:1 with PMAi exposure. We characterized treated suppressed HIV-1 infection as a period of inefficient, not absent, virus transcription. Multiply spliced HIV-1 transcripts and virion production did not consistently increase with LRA boosting, suggesting the presence of a persistent posttranscriptional block. LRA boosting can be leveraged to probe mechanisms of an effective cellular HIV-1 latency reversal program.

## Abstract 2

HIV-1 latency reversal agent (LRA) boosting can maximize HIV-1 cell-associated RNA levels and identifies novel immunology and virus transcription biology in people with HIV.

## Introduction

HIV-1 proviral DNA remains integrated in CD4^+^ T cells during suppressive antiretroviral therapy (ART) and is a barrier to cure. This virus reservoir, defined as cells that contain provirus capable of generating infectious virions, is initially seeded within days of infection before symptoms develop (1, 2), persists (3, 4), and can be maintained indefinitely (5). The best approach to pursue HIV eradication remains uncertain. One strategy aims to pharmacologically reactivate latent HIV transcription in the setting of suppressive ART, induce virus protein production in infected cells, and then clear the reservoir through immune-mediated mechanisms (6–8). HIV-1 latency reversal agents (LRAs) have advanced into clinical trials as monotherapy but with modest results; transient increases in virus cell-associated RNA (caRNA) production may be observed with some LRAs but does not translate into a decrease in reservoir size (9–17). HIV-1 latency may be characterized by transcriptional blocks that cannot be overcome by a single LRA (18–21).

To address these limitations, novel classes and combinations of LRAs have been studied (22). Epigenetic LRAs such as histone deacetylase inhibitors (HDACi), when combined with PKC agonists or second mitochondria-derived activator of caspases (Smac) mimetics, have been reported under some conditions to synergistically increase HIV-1 transcription in CD4^+^ T cells isolated from virally suppressed participants with HIV (23–27). Generally, the sample sizes were small and evaluated LRA concentrations in the mid-nanomolar to micromolar range. The absolute amount of HIV-1 latency reversal reported with combination LRA, defined primarily as induced levels of HIV-1 caRNA, has been significantly less than that observed with the maximal HIV-1 reactivating combination of PMA and ionomycin (PMAi). A trade-off may exist between maximum synergy and maximum efficacy in combination LRA approaches (28).

While HIV-1 is generally accepted to be transcriptionally silent during suppressive ART, this latency may not be absolute. Studies demonstrate that short promoter proximal transcripts may be generated in the absence of HIV-1 Tat (29, 30). Resting CD4^+^ T cells isolated from people with HIV (PWH) may contain transcripts extending beyond the transcriptional pausing site, a minority of which may be full-length or spliced and polyadenylated [poly(A)] (20, 31). Total CD4^+^ T cells isolated from PWH may contain approximately 7-fold more proximal HIV-1 transcripts, of at least 185 nucleotides in length, than poly(A) virus mRNA (18). HIV-1 caRNA is readily detectable in blood and tissue of PWH on virologically suppressive ART (32–35).

PWH may be exposed to opioids, whether prescribed for chronic pain (36), prescribed for opioid use disorder (37), or injected as heroin (38). The pharmacology of opioids associated with substance use disorders may differ from opioid medications used to treat chronic pain. The μ opioid agonists, e.g., heroin, fentanyl, morphine, and methadone, can inhibit transcription factors known to be positive regulators of HIV transcription initiation (39–41). The effects of buprenorphine, a partial μ opioid agonist and κ and δ opioid antagonist commonly used to treat opioid use disorder, on HIV-1 latency reversal have not been explored. PWH who use opioids may have diminished responses to latency reversal mediated by TCR agonism (42).

To investigate the mechanisms controlling HIV-1 latency reversal during opioid use, we enrolled a cohort of 36 treated, virologically suppressed participants with HIV. We originally hypothesized that opioid use would limit HIV-1 latency reversal and vary as a function of opioid use type. We tested LRA combinations at concentrations lower than those typically assessed and identified the phenomenon of LRA boosting: synergistic HIV-1 transcriptional reactivation with small molecules that do not reverse latency as single agents. We corroborated these findings in an additional 11 participants from a separate cohort and identified key associations between potent LRA boosting, increases in histone acetylation, modulation of T cell phenotype and function, and downstream blocks to virion production. While benchmarking LRA boosting, we observed that HIV-1 transcription during treated infection may be greater than previously appreciated and varies by CD4^+^ T cell phenotype. Importantly, we find that opioid use did not limit the magnitude or mechanisms of LRA boosting.

## Results

### Opioid use does not affect HIV-1 reservoir size.

We enrolled the Opioids, HIV, and Translation (OPHION) cohort from a single academic center of 36 virally suppressed PWH who used, or did not use, opioids in a 2:1 ratio. Participants had a median age of 59 years, included one-third women, and were approximately 30% White (Table 1). The median ART duration was 12 years with a median duration of undetectable plasma HIV-1 RNA levels of over 6 years. Participant characteristics were further defined by type of opioid use (Supplemental Table 1; supplemental material available online with this article; https://doi.org/10.1172/jci.insight.185480DS1) and validated by urine toxicology and self-reported substance use questionnaires (Supplemental Figure 1). To reflect the range of opioid use encountered in clinical practice, this cohort comprised participants who actively injected opioids (*n* = 4), used methadone (*n* = 4) or suboxone (*n* = 12) as medication for opioid use disorder, or took opioids for chronic pain (*n* = 4). Amphetamine and barbiturate use were not detected. All active injection opioid users had confirmatory urine toxicology screens positive for fentanyl (*n* = 2 of 4, range 62–>500 ng/mL) and/or norfentanyl (*n* = 4 of 4 participants, range 46–>500 ng/mL) and reported a median of 6 days of injection opioid use in the preceding 30 days (IQR 4–16 days) with a median of 7 days since the most recent use (IQR 4–14 days). All participants in the methadone, buprenorphine, and prescription opioid subgroups had urine toxicology positive for that substance.

**Figure 1:**
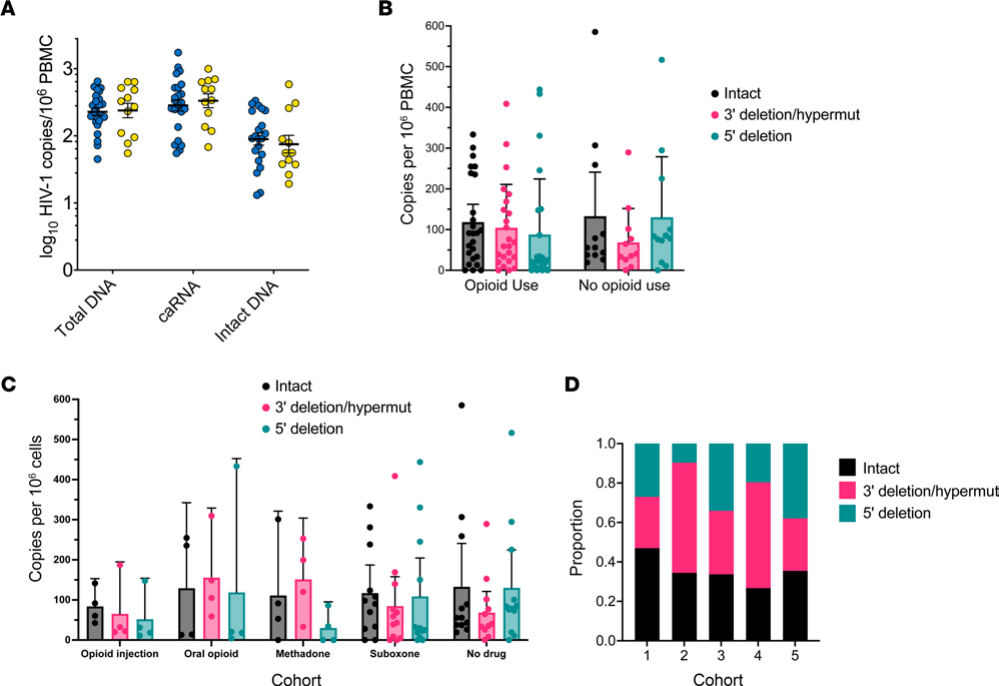
HIV-1 reservoir size and intactness in participants who used (*n* = 24) or did not use (*n* = 12) opioids. (**A**) HIV-1 persistence measures in participants with (blue circles) and without (yellow circles) opioid use are shown. Statistical significance was assessed with Wilcoxon’s signed-rank tests. (**B**) 5′ and 3′ genomic deletions as a function of opioid use. Mean ± 95% CIs are displayed. (**C**) Opioid subgroup analysis. The reservoir sizes of intact, 5′-deleted, and 3′-deleted or -hypermutated regions are shown. (**D**) The proportion of intact genomes as a function of DNA copies and opioid use subgroup; Kruskal-Wallis testing adjusting for multiple comparisons was not significant. Cohort 1, injection opioid use; cohort 2, prescribed oral opioids for pain; cohort 3, methadone; cohort 4, suboxone; cohort 5, no opioid use.

To assess markers of HIV-1 persistence, we quantified total HIV-1 DNA and caRNA in PBMCs and evaluated intact proviral reservoir size (Figure 1). Total HIV-1 DNA levels were statistically similar between opioid and nonopioid groups, 2.36 versus 2.38 log_10_ copies/10^6^ PBMCs, respectively, as were HIV-1 caRNA levels, 2.45 versus 2.52 log_10_ copies/10^6^ PBMCs (Figure 1A). Levels of intact provirus DNA were also similar between groups, 2.00 versus 1.82 log_10_ copies/10^6^ PBMCs. To better define the intact proviral DNA reservoir, we characterized 5′ and 3′ HIV-1 deletions across cohorts and opioid use subgroups (Figure 1, B and C). Similar proportions of intact 5′ and 3′ genetic regions were observed, irrespective of opioid use. The ratios of proviruses containing an intact 5′ region and a defective (hypermutated) or deleted 3′ region in HIV-1 *env* were statistically similar. An exploratory analysis demonstrated a greater proportion of total genomes were intact in active injection opioid users, though this result was not significant when adjusted for multiple comparisons (Figure 1D).

### LRA boosting markedly increases HIV-1 transcription.

**Figure 2:**
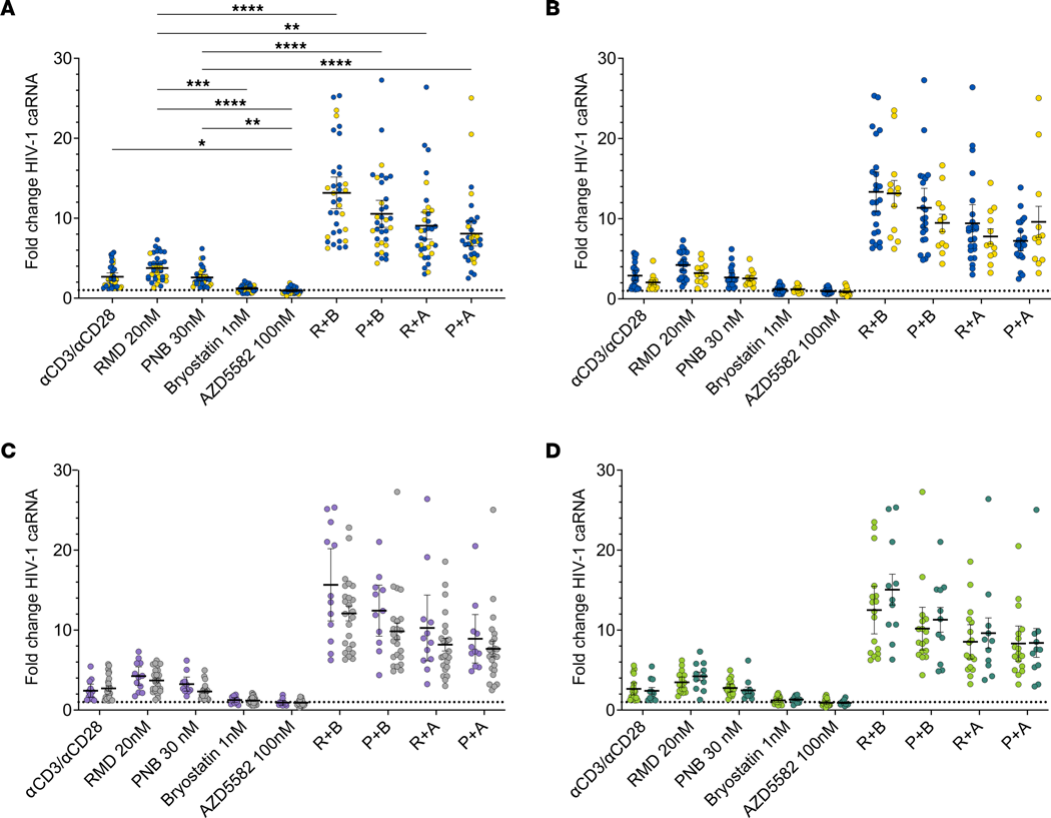
HIV-1 latency reversal in OPHION participants. HIV-1 caRNA levels were quantified after 24-hour incubations; mean values and 95% CIs are shown. (**A**) Combined data showing LRA responses in 36 total participants with (blue circles) or without (yellow circles) opioid use. Data from **A** were dichotomized by (**B**) opioid use, (**C**) sex (light purple circles, male; light gray circles, female), and (**D**) race (light green circles, Black and American Indian; dark green circles, white). Means and SEM are shown. Dotted horizontal line denotes a fold-change of 1. *P < 0.05, ** *P* < 0.01, *** *P* < 0.001, and **** *P* < 0.0001 for Kruskal-Wallis testing, corrected with Dunn’s multiple-comparison test. αCD3/αCD28, anti-CD3 anti-CD28 superparamagnetic beads; RMD, romidepsin; PNB, panobinostat; R, RMD 20 nM; P, PNB 30 nM; B, bryostatin 1 nM; A, AZD5582 100 nM.

To determine the effects of opioid use on HIV-1 latency reversal ex vivo, we isolated PBMCs from 36 participants in the OPHION cohort and tested 10 LRA conditions (Figure 2). The HDACi RMD and PNB were used at the lowest concentrations most commonly reported in the literature, and the PKC agonist bryostatin was tested at 1 nM, one-tenth the most commonly reported concentration. In single LRA conditions, we observed a 2.7-fold activation of HIV-1 caRNA transcription, relative to an untreated DMSO-containing control, with anti-CD3/anti-CD28 (αCD3/αCD28) beads, a TCR agonist (Figure 2A). This magnitude of TCR agonism–induced latency reversal is consistent with prior studies (18, 43–47). Statistically greater reactivation was observed with RMD (3.8-fold increase) when compared with PNB (2.6-fold increase) (Supporting Data Values). Low-dose bryostatin minimally increased unspliced HIV-1 caRNA levels (1.2-fold increase), but the Smac mimetic AZD5582 (AZD), at standard concentration (48), did not (0.9-fold increase, 95% CI 0.8, 1.1).

We next assessed LRAs in combination. Incubation of PBMCs with HDACi and either AZD or low-dose bryostatin potentiated HIV-1 latency reversal. AZD in combination with PNB or RMD increased HIV-1 caRNA levels 8.1-fold and 9.1-fold, respectively. Low-dose bryostatin with PNB increased HIV-1 caRNA 10.6-fold, whereas the greatest fold-induction of HIV-1 caRNA was observed with the combination of low-dose bryostatin and RMD (13.2-fold). Statistically greater HIV-1 RNA induction was observed with the LRA boosting combinations relative to HDACi monotherapy and TCR agonism, when corrected for multiple comparisons. We compared LRA response as a function of opioid use (Figure 2B), sex (Figure 2C), race (Figure 2D), and ethnicity (Supplemental Figure 2A) and observed no statistically significant differences in the fold-changes of HIV-1 caRNA induction across these groups. In an exploratory analysis, the fold-change in HIV-1 caRNA varied by opioid use subgroup in response to TCR agonism (Supplemental Figure 2B). Statistically significantly greater reactivation with αCD3/αCD28 beads was observed in the suboxone subgroup when compared with active injection opioid users, the group with the smallest fold-change in TCR agonism-induced HIV-1 caRNA levels.

### LRA boosting modulates T cell activation and cytokine production.

**Figure 3:**
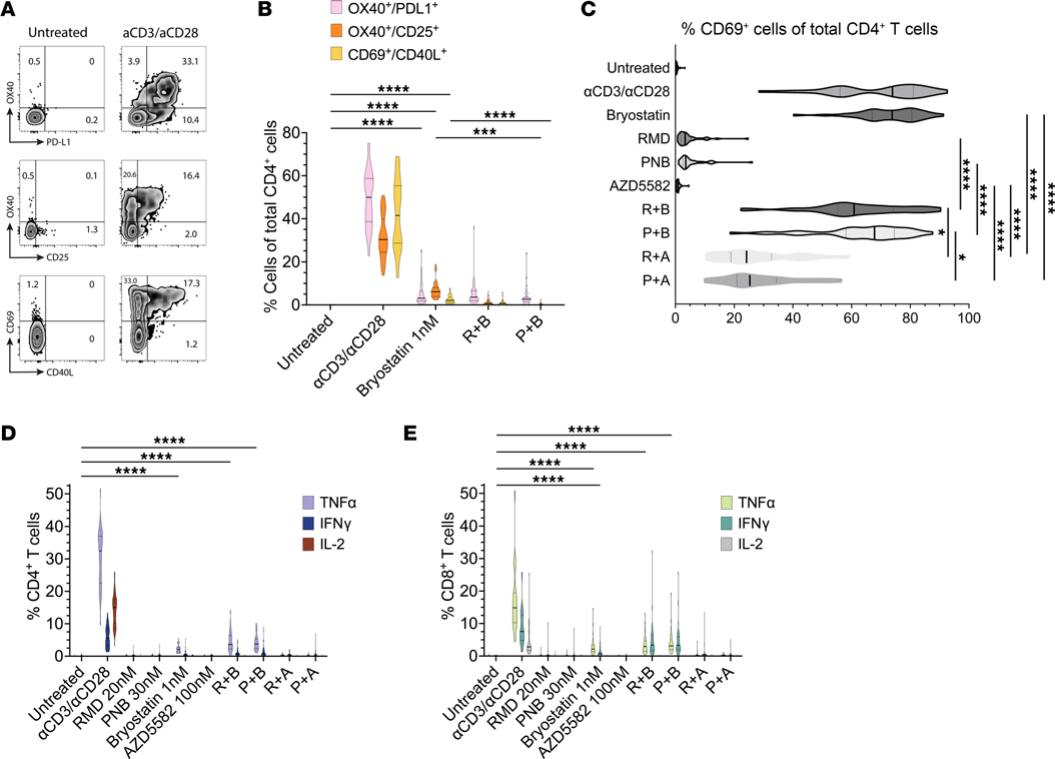
Immune activation and cytokine induction during LRA boosting. (**A**) Example plots of OX40^+^PD-L1^+^, OX40^+^CD25^+^, and CD69^+^CD40L^+^ expression from gated live CD4^+^ T cells in PBMC samples incubated with DMSO control or an αCD3/αCD28 bead-positive control for 18 hours. (**B**) Violin plots of surface AIM response as a function of LRA. Results across 3 AIM assays for OPHION participants’ samples (*n* = 36) are shown for comparison. (**C**) Quantification of LRA-induced surface CD69 expression in live total CD4^+^ T cells (*n* = 36). Intracellular production of the cytokines TNF-α, IFN-γ, and IL-2 during LRA exposure in live (**D**) CD4^+^ and (**E**) CD8^+^ T cells (*n* = 36). Violin plot median values are indicated by a solid black line; quartiles are shown with a dotted line. LRA conditions without visible bars represent values < 0.1% of cells. *P < 0.05, *** *P* < 0.001, and **** *P* < 0.0001 for Kruskal-Wallis, corrected with Dunn’s multiple-comparison test. R, RMD 20 nM; P, PNB 30 nM; B, bryostatin 1 nM; A, AZD5582 100 nM.

To understand whether LRA boosting activates CD4^+^ T cells, we assessed the upregulation of surface activation-induced markers (AIMs) (49). We leveraged dual-marker AIM assays (Supplemental Figure 3 and Figure 3A), originally developed to detect T cell antigen responsiveness, to quantify cellular activation that occurs with LRA exposure, outside the context of peptide-specific recognition. Using TCR agonism as a positive control, responses were detected in 49%, 32%, and 42% of CD4^+^ T cells in the OX40/PDL1, OX40/CD25, and CD69/CD40L AIM assays, respectively (Figure 3B). Low-dose bryostatin significantly upregulated surface expression to more modest levels in all 3 AIM assays, relative to an untreated control condition. Bryostatin induced OX40/PD-L1 surface expression in 5.2% of CD4^+^ T cells, levels of induction that did not significantly change when used in combination with RMD (5.4%) or PNB (3.5%). Bryostatin monotherapy induced expression in 6.9% and 2.6% of CD4s in the OX40/CD25 and CD69/CD40L AIM assays, respectively. In these 2 assays, the combination of RMD or PNB with low-dose bryostatin significantly reduced AIMs, relative to bryostatin alone and in some cases to control (untreated) levels; these findings did not differ by opioid use (Supplemental Figure 4, A–C). The remainder of the LRA panel and their combinations did not upregulate surface expression in any AIM assay.

We observed a more pronounced upregulation in isolated CD69 expression, an early activation marker, in response to LRAs. Approximately 0.5% of untreated CD4^+^ T cells expressed CD69 after 18 hours in culture (Figure 3C). The proportion of cells expressing surface CD69 significantly increased to similar levels with TCR agonism (68.5%) and low-dose bryostatin (72.9%). Whereas AZD5582 (1.0%), RMD (4.7%), and PNB (5.4%) modestly increased CD69 levels, combinations of these small molecules led to synergistic effects. Significantly greater induction of CD69 expression was observed when RMD or PNB was combined with bryostatin or AZD; a bryostatin combination induced more CD69 than an HDACi plus AZD. CD69 levels were statistically similar when comparing TCR agonism, bryostatin alone, and bryostatin in combination with either HDACi (Supplemental Figure 4D).

To further probe LRA effects on T cell activation, we stained CD4^+^ (CD4s) and CD8^+^ T cells (CD8s) for intracellular cytokine production (Figure 3, D and E). Whereas cytokine assessments in T lymphocytes most commonly include IL-2 and IFN-γ, we additionally quantified intracellular TNF-α by flow cytometry. We observed a cytokine production hierarchy in CD4s, where the levels were TNF-α > IL-2 > IFN-γ, and CD8s, where TNF-α > IFN-γ > IL-2. In response to TCR agonism, significantly more CD4s produced TNF-α (30.4%) than IL-2 (14.3%) or IFN-γ (6.0%) and significantly more IL-2 than IFN-γ (Supplemental Figure 4E). We identified a different hierarchy in CD8s, where the proportion of cells producing TNF-α remained greatest (17.5%), but significantly more IFN-γ (9.0%) was produced compared with IL-2 (4.3%). Whereas HDACi monotherapy did not increase the proportion of CD4s or CD8s producing cytokines compared with an untreated control, low-dose bryostatin alone or in combination with HDACi significantly increased production of TNF-α and, to a smaller magnitude, IFN-γ in CD4s (*P* < 0.001 for each comparison). Significantly more CD4s produced TNF-α than IFN-γ in response to RMD + bryostatin (4.5% versus 1.3%) and PNB + bryostatin (4.2% versus 1.7%) (Supporting Data Values). In CD8s, low-dose bryostatin significantly increased production of TNF-α (2.8%) and IFN-γ (1.5%). Proportions of CD8s that produced TNF-α and IFN-γ when exposed to the combinations of RMD + bryostatin (3.4% versus 4.7%) and PNB + bryostatin (4.1% versus 4.6%) were similar and were statistically similar to bryostatin alone. IL-2 production did not increase above 0.1% of CD4s or CD8s for any LRA or combination.

### LRA boosting increases histone acetylation.

**Figure 4:**
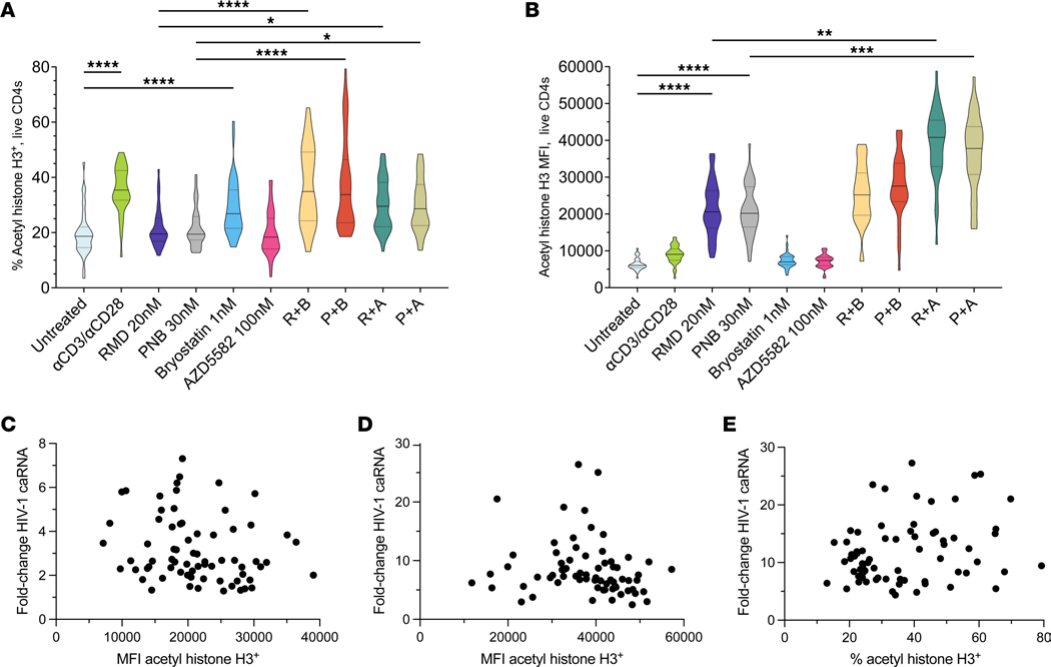
LRA boosting increases histone acetylation. (**A**) Violin plots showing the proportion of gated live total CD4^+^ T cells with acetylated histone H3, assessed by flow cytometry. (**B**) Violin plot of MFI of acetylated histone H3 per live CD4. Scatterplots of acetylated histone H3 levels per CD4 as a function of HIV-1 transcription in response to RMD and PNB, (**C**) LRA boosting with HDACi and AZD5582 (**D**), and HDACi in combination with low-dose bryostatin (**E**). Acetylated histone levels and HIV-1 reactivation with the combinations of HDACi and bryostatin were moderately positively correlated (*r* = 0.25, *P* = 0.04). Violin plot solid black line, median values; quartiles are shown with a dotted line. *P < 0.05, ** *P* < 0.01, *** *P* < 0.001, and **** *P* < 0.0001 for Kruskal-Wallis testing, corrected with Dunn’s multiple-comparison test.

To investigate the mechanism(s) of LRA boosting, we next assessed histone acetylation. The proportions of acetylated histone H3^+^ live CD4s were similar between untreated cells (19.7%) and cells exposed to RMD (21.8%), PNB (21.7%), or AZD (19.8%) and significantly increased after single-LRA exposure to αCD3/αCD28 beads (35.5%) and low-dose bryostatin (29.2%) (Figure 4A). Low-dose bryostatin in combination with either RMD (36.8%) or PNB (37.5%) significantly increased the proportions of CD4s containing acetylated histone H3. Interestingly, statistically significant increases in proportions of acetylated histone H3^+^ live CD4s were also observed when combining LRA, which individually did not affect these percentages, specifically RMD + AZD (30.0%) and PNB + AZD (29.8%). These results did not differ by opioid use (Supplemental Figure 5A).

Levels of acetylated histone H3, measured by MFI, were similar in untreated live CD4s and in cells cultured with αCD3/αCD28 beads, low-dose bryostatin, or AZD (Figure 4B). Significant 3.4-fold and 3.3-fold increases in acetyl histone MFI were observed for RMD and PNB, respectively. AZD in combination with RMD or PNB further increased MFI by 6.1-fold and 5.7-fold, respectively. Absolute increases in MFI were observed for low-dose bryostatin in combination with RMD or PNB but did not retain significance after correction for multiple comparisons. These results did not differ by opioid use (Supplemental Figure 5B). We observed no association between acetylated histone H3 MFI and fold-change in HIV-1 RNA levels for HDACi monotherapy or for HDACi in combination with AZD (Figure 4, C and D). A statistically significant weak association between the proportion of acetyl histone H3^+^ CD4s and fold-change in HIV-1 RNA levels was detected for HDACi in combination with bryostatin (Figure 4E).

### LRA boosting does not consistently induce virion production.

**Figure 5:**
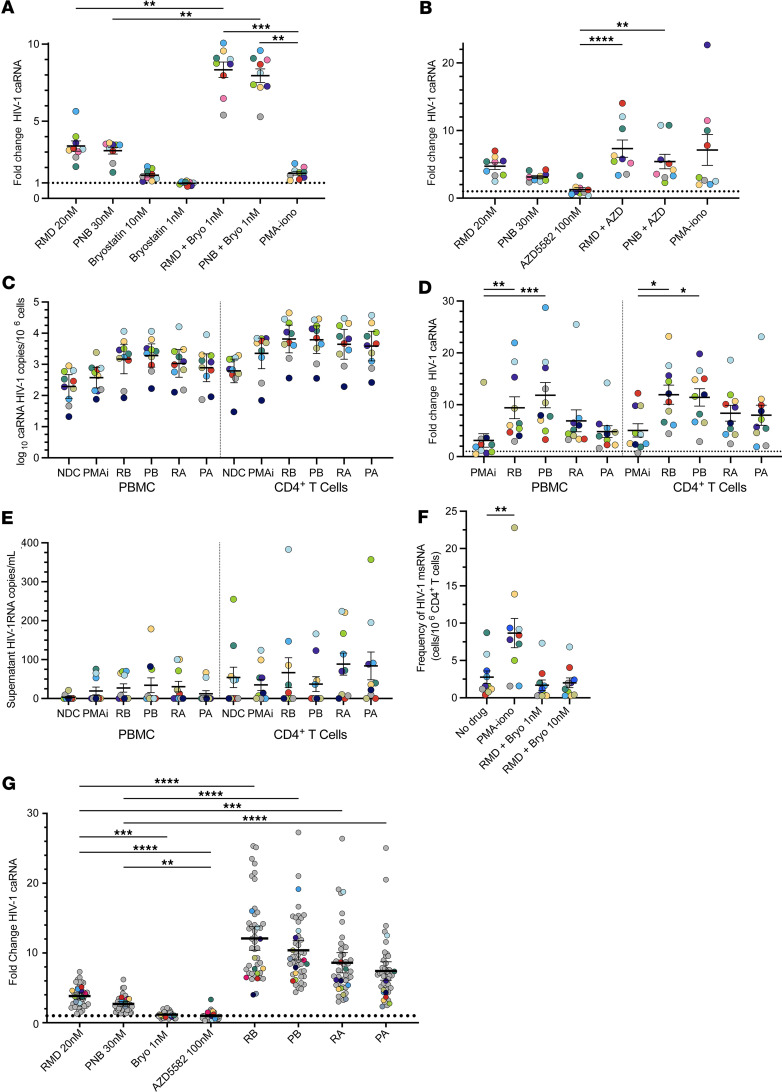
Comparative analysis of LRA boosting effects in PBMCs and CD4^+^ T cells. HIV-1 unspliced caRNA levels were assessed in HEAL cohort participant samples (*n* = 11) during (**A**) low-dose bryostatin-based and (**B**) AZD5582-based LRA boosting combinations in PBMCs, when compared with PMAi. Data are presented as means ± SEM. Circle color denotes a given HEAL participant across figure panels. (**C**) Absolute HIV-1 unspliced caRNA levels in parallel PBMC and CD4^+^ T cell LRA exposures. PBMC and CD4 results are separated by a vertical dotted line. (**D**) Representation of data in **C** as fold-change in HIV-1 caRNA levels. (**E**) Corresponding HIV-1 RNA levels from culture supernatants. (**F**) Multiply spliced HIV-1 RNA levels as determined in a modified TILDA, comparing RMD-bryostatin combinations at 2 bryostatin concentrations and PMAi. (**G**) Combined OPHION and HEAL participant results (*n* = 47) that summarize PBMC LRA boosting responses, for LRA conditions common to both datasets. Results from OPHION participant samples, carried over from Figure 2A, are shown in gray circles. HEAL participant results are shown in color. *P < 0.05, ** *P* < 0.01, *** *P* < 0.001, **** *P* < 0.0001 for Kruskal-Wallis, corrected with Dunn’s multiple-comparison test. NDC, no-drug control, contains 0.2% DMSO; R, RMD 20 nM; P, PNB 30 nM; B, bryostatin 1 nM; A, AZD5582 100 nM.

To extend our analyses from the OPHION cohort, we next studied an additional 11 participants with HIV who did not use opioids, in the HIV Eradication and Latency (HEAL) cohort, for whom leukapheresis samples were available (Supplemental Table 2). We compared LRA boosting responses in PBMCs with the most potent HIV-1 LRA, PMAi. Significant fold-change increases in HIV-1 transcription were observed with combinations of low-dose bryostatin with RMD (8.3-fold) or PNB (8.0-fold), when compared with RMD alone (3.4-fold), PNB alone (3.1-fold), or PMAi (1.6-fold) (Figure 5A). We observed statistically similar HIV-1 transcription with an HDACi in combination with AZD, relative to PMAi (Figure 5B). No significant increases in HIV-1 transcription were observed in PBMC experiments with low-dose bryostatin (1.0-fold, 95% CI 0.9, 1.1) or AZD (1.2-fold, 95% CI 0.6, 1.9). Similar magnitudes of HIV-1 transcriptional reactivation were seen with HDACi monotherapy, low-dose bryostatin, and AZD monotherapy across the OPHION and HEAL cohorts we studied. We evaluated human reference gene transcription per million PBMCs during LRA treatment and observed decreases with HDACi exposure, increases with PMAi, and variable changes with bryostatin (Supplemental Figure 6) (32, 50).

To assess the magnitude of HIV-1 latency reversal as a function of cell type, we next performed parallel experiments in PBMCs and total CD4s (Figure 5C). While CD4s are the only established HIV-1 reservoir in PBMCs, we performed this comparison across cell types to increase experimental rigor. Levels of HIV-1 caRNA were, on average, 4.3-fold higher (range 3.2–6.0) across experimental conditions performed in CD4s, when compared with PBMCs, and varied by 1.6 log_10_ (PBMC) and 1.8 log_10_ (CD4) across participant samples in the absence of LRA exposure. To control for absolute caRNA differences, we determined fold-changes in LRA response and observed statistically significant increases in HIV-1 caRNA with low-dose bryostatin in combination with RMD or PNB, when compared with PMAi, in both PBMCs and CD4s (Figure 5D). To explore the variance in LRA response, we compiled per-participant biological replicate measurements (Supplemental Figure 7A). Supernatant HIV-1 RNA levels increased after 24-hour incubation of PBMCs with PMAi (19 copies/mL, 95% CI 10, 32) and the combinations of RMD plus bryostatin (27 copies/mL, 95% CI 11, 34) or AZD (31 copies/mL, 95% CI 14, 43), and PNB plus bryostatin (35 copies/mL, 95% CI 19, 60) or AZD (12 copies/mL, 95% CI 8.1, 26), when compared with untreated PBMCs (3.0 copies/mL, 95% CI 2.2, 6.9), but these results were not statistically significant after correction for multiple comparisons (Figure 5E). This virion production response was dichotomized by participant. For each LRA condition, PBMC samples from at least 5 of 10 participants showed no increases in HIV-1 RNA levels over control; these were most commonly the same participants. Additionally, no significant increases in LRA-induced virion production in isolated CD4^+^ T cell cultures were observed. Greater virion production in untreated CD4s and greater absolute magnitudes of LRA-associated supernatant viremia in CD4s were noted, relative to PBMCs, but these increases were seen in only a subset of participants. To define this posttranscriptional block more precisely, we quantified the frequency of CD4s with inducible multiply spliced HIV-1 RNA transcripts (Figure 5F) (51). No significant increases in the frequency of cells that contain HIV-1 *tat*/*rev* transcripts were observed with RMD in combination with bryostatin at either 1 nM or 10 nM concentration. In a post hoc analysis, we combined datasets from OPHION and HEAL participants, including LRA conditions common to both sets of experiments and observed statistically significant increases in HIV-1 unspliced RNA transcription with all LRA boosting combinations (Figure 5G). The variance in LRA response, defined by the IQR, increased as the magnitude of fold-change in HIV-1 caRNA increased (Supplemental Figure 8).

### Effective latency reversal normalizes unspliced/poly(A) HIV-1 mRNA ratios.

**Figure 6:**
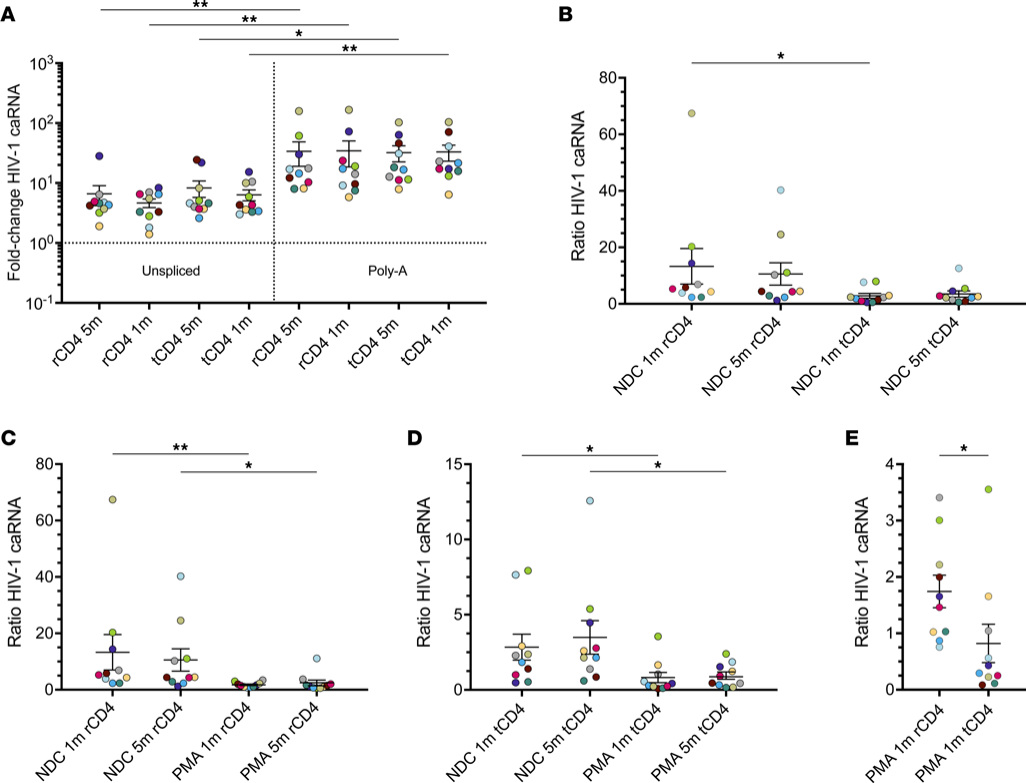
Effective latency reversal normalizes unspliced/polyadenylated HIV-1 mRNA ratios. (**A**) Resting and total CD4^+^ T cell HIV-1 transcriptional profiles during PMAi exposure. HEAL participant samples (*n* = 10) were used to assess the effects of CD4 phenotype and cell culture density on fold-changes in HIV-1 unspliced and poly(A) transcripts during latency reversal with PMAi. (**B**) Ratios of HIV-1 unspliced and poly(A) transcripts in resting and total CD4^+^ T cells isolated from PWH. The effects of PMAi and cell density on HIV-1 latency reversal in (**C**) resting CD4^+^ T cells and (**D**) total CD4^+^ T cells and (**E**) the specific ratios of HIV-1 caRNA production in rCD4 compared with tCD4 when plated at a density of 1 million cells/mL. A given HEAL participant is indicated by circle color. Ratios of HIV-1 caRNA are the ratios of unspliced HIV-1 caRNA to poly(A) HIV-1 mRNA. *P < 0.05, ** *P* < 0.01, for Kruskal-Wallis, corrected with Dunn’s multiple-comparison test in **A**–**D**. Panel **E** was assessed with a Wilcoxon’s matched pairs signed-rank test. PMA, PMA 50 ng/mL with ionomycin 1 μM; Poly-A, polyadenylated HIV-1 caRNA; unspliced, HIV-1 unspliced caRNA; 1m, 1 million cells/mL culture media; 5m, 5 million cells/mL culture media.

We observed lower PMAi-associated fold-changes in HIV-1 caRNA levels with our unspliced HIV-1 RNA quantitative PCR (qPCR) assay when compared with prior studies quantifying poly(A) HIV-1 mRNA (23, 27). These assays differ methodologically, so we directly compared them and assessed the effects of primer location, CD4 phenotype, and CD4 density on HIV-1 caRNA quantifications (Figure 6A). In response to a 24-hour PMAi exposure, levels of HIV-1 unspliced caRNA increased 4.3- to 8.6-fold. These levels did not significantly differ between resting (rCD4, CD25^–^CD69^–^HLA-DR^–^) and total CD4^+^ T cell (tCD4) stimulations or when CD4s were cultured at densities of 5 × 10^6^ or 1 × 10^6^ cells/mL. Poly(A) HIV-1 caRNA levels increased 32- to 34-fold with the same PMAi stimulation and were statistically significantly greater than unspliced HIV-1 caRNA fold-changes for each input CD4^+^ T cell phenotype or cell density (*P* < 0.01, Figure 6A). While PMAi-induced fold-changes in HIV-1 RNA were similar between rCD4 and tCD4, we observed differences in the amounts of unspliced and polyadenylated message present in these untreated cell populations. In the absence of LRA treatment, significantly greater ratios of unspliced to poly(A) HIV-1 caRNA were seen in rCD4 (10.6–13.3), relative to tCD4 (2.8–3.5) (Figure 6B). The differences in these ratios were driven by differences in HIV-1 poly(A) transcript levels. While untreated rCD4 and tCD4 at 5 million or 1 million cells/mL contained similar absolute levels of unspliced HIV-1 caRNA transcripts (*P* = 0.98 for each), untreated tCD4 demonstrated statistically greater copy numbers of HIV-1 poly(A) mRNA per million CD4s (*P* < 0.005 at 5m cells/mL, *P* < 0.001 at 1m cells/mL, Freidman’s test [1-way ANOVA] with Dunn’s correction for multiple comparisons; Supporting Data Values). PMAi stimulation normalized unspliced/poly(A) ratios to 1.7–2.4 in rCD4 and 0.8–1.0 in tCD4s (Figure 6, C and D). Significantly lower ratios of unspliced/poly(A) HIV-1 RNA were observed in reactivated tCD4, when compared with rCD4 (Figure 6E).

## Discussion

An approach to cure HIV infection should be equally effective for all people. Here we explored how opioid use may affect HIV-1 latency and its reversal. Opioid use is complex and can be accompanied by comorbidities, such as immune activation with injection drug use (52, 53), that may affect the HIV-1 reservoir and its persistence (54–56). Here we found no effects of opioid use by indication, type, or pharmacology on markers of HIV-1 persistence. The HIV-1 reservoir sizes we observed are consistent with prior work (32, 57). In persistently suppressed viremia, opioid use was less likely to modulate virus reservoir size, in agreement with recent studies of injection heroin use (42, 58). Overall, participants were matched on the duration of ART and virus suppression, key variables associated with HIV-1 reservoir size (57), and our urine toxicology results were consistent with the participants’ ascribed opioid use group.

We used samples from opioid users to identify the phenomenon of LRA boosting: improving the potency of HDACi beyond that of PMAi by combining with a second drug that does not, by itself, reactivate HIV-1 transcription. This boosting occurred at lower LRA concentrations, here at least half the RMD, 1/10th the bryostatin, and 1/100th the Smac mimetic concentrations used in prior combination studies (23–26). Here we leveraged samples from 47 participants to confirm those findings, extend the observation to HDACi in combination with low-dose bryostatin more generally, and identify LRA boosting with a Smac mimetic. A recent study identified that injection opioid use was associated with a lack of ex vivo HIV-1 activation to TCR agonism (42); our findings in the subgroup of active injection opioid users agree. However, this observation did not extend to other types of opioid use, e.g., suboxone use. Additional studies should corroborate these findings in a larger sample and investigate why HIV-1 latency reversal by TCR agonism may be particularly affected by injection opioids.

We observed no AZD5582-associated HIV-1 latency reversal activity when used as a single agent in PBMCs isolated from 47 participants. AZD5582 monotherapy has been assessed for human ex vivo HIV-1 latency reversal in prior studies (48, 59–61). In these studies, QVOA and p24 responses were not consistently observed in the majority of participants. Our findings agree more with recent work that did not identify AZD5582 monotherapy–associated HIV-1 LRA activity (27); these 2 studies are characterized by larger sample sizes and uniform statistical analyses.

Our relatively large sample size, for this field, allowed us to determine that LRA boosting was less likely to be affected by sex, race, or ethnicity. We do note, however, that nonopioid participants in our OPHION and HEAL cohorts were more likely to be Black, whereas participants who used opioids were a more racially and ethnically mixed population. Similar magnitudes of boosting were observed with RMD and PNB and suggests that class I histone deacetylase inhibition may be more relevant to this phenomenon (63, 64).

We found that HDACi monotherapy increases the amount of acetylated histone H3 per CD4 but not the proportions of acetylated histone H3^+^ CD4s. HDACi-induced changes in acetylated histone (AcH) H3 and/or H4 are nearly exclusively reported in the literature as changes in MFI (10, 13–16, 64–67), not as a function of the percentage AcH-positive cells (13). To increase transparency, we report histone acetylation changes both as the proportion of AcH-positive cells and by MFI; the 3- to 4-fold MFI increases we report with RMD and PNB are consistent with prior literature. LRA boosting correlated with increased histone H3 acetylation in CD4^+^ T cells, but AZD5582-associated boosting did not recruit additional CD4s with AcH H3. In the context of cellular genes, histone acetylation in some cell types may not be an immediate driver of the transcriptional changes observed with HDACi (68).

Similar magnitudes of HIV-1 reactivation were observed with low-dose bryostatin and AZD5582-mediated HDACi boosting, suggesting the possibility of shared mechanisms. Bryostatin and AZD5582 can activate canonical and noncanonical NF-κB pathways, respectively, and how HIV-1 latency reversal integrates this signaling requires further definition. A recent study that performed an AZD5582-stimulated CRISPR screen identified HDAC2 as a potential synergistic drug target (27). To assess the generalizability of the LRA boosting effects we observed with bryostatin, additional PKC agonists and canonical NF-κB signaling LRA should be tested.

We found that HIV-1 latency during treated infection in PWH is best described as a period of inefficient, but not absent, virus mRNA production in blood (31, 69, 70). qPCR assays that quantify HIV-1 persistence markers are not necessarily standardized across research groups; the fold-change in HIV-1 unspliced caRNA levels that we observed with PMAi stimulation was an order of magnitude lower than some prior studies, which quantified polyadenylated HIV-1 mRNA (23, 27). Our findings suggest this difference is not a result of assay technical performance. Rather, qPCR assays of HIV-1 unspliced and poly(A) caRNA levels may capture distinct biology, as a function of CD4 phenotype. First, we identified tCD4 contain more polyadenylated HIV-1 message than rCD4 in PWH. Second, PMAi stimulation has more pronounced effects on the induction of HIV-1 poly(A) mRNA levels, relative to unspliced RNA, and explains the discrepancy in PMAi-induced HIV-1 caRNA fold-changes between our study and others. Third, PMAi stimulation increases the levels of both HIV-1 unspliced and poly(A) and, in doing so, normalizes their ratios; this normalization is greater in tCD4. Latency reversal results in greater fold-change increases to HIV-1 poly(A) mRNA than unspliced message.

Based on the location of our HIV-1 caRNA qPCR primers, we identify that during treated virologically suppressed infection, HIV-1 transcription generates unspliced transcripts of at least 350 nucleotides in length. We note this is distal to where RNA polymerase II promoter–proximal pausing typically occurs; to previously described short, abortive HIV-1 transcripts; and to the proximal HIV-1 transcripts identified by droplet digital PCR–based approaches (18, 30, 31, 71, 72). We conclude that the efficiency of HIV-1 transcription is therefore reduced during ART but not extinguished. We speculate that while the HIV-1 long terminal repeat can recruit RNA polymerase II during ART, the transcription elongation complex may be missing virus and/or host factors required to maximize virus mRNA production and processing.

LRA boosting, and their individual components, can have off-target effects. We used cell surface and intracellular assessments of immune activation to investigate whether LRA boosting can be uncoupled from immune activation. Whereas CD69 surface expression has been proposed as a biomarker of LRA potency in the context of PKC agonists, we found no correlation between CD69 upregulation and the magnitude of latency reversal (73, 74). We identified less induction of CD69 expression with RMD monotherapy than a prior study, despite similar staining and gating strategies (75). While low-dose bryostatin was sufficient to increase surface activation-induced markers and increase cytokine production in CD4^+^ and CD8^+^ T cells, albeit to relatively low levels, the increased HIV-1 reactivation seen with LRA boosting did not increase surface AIM and in some cases reduced it — specifically by adding an HDACi to bryostatin. However, intracellular cytokine production increased modestly with these same combinations and suggests that AIM assays and intracellular cytokine staining provide complementary information in the assessment of investigational LRA. Our findings suggest that future studies of LRA-induced cytokine production include TNF-α quantifications, whose levels are more likely to increase than either of the more commonly assessed IL-2 and IFN-γ. In contrast, LRA boosting with AZD5582 resulted in relatively lower surface levels of CD69 and no induction of surface activation markers or cytokine production in T cells. Our results extend previous findings on the effects of combination LRA on CD8^+^ T lymphocytes to CD4s (76) and suggest that cytokine assessments of IL-2 production in CD4s provide the least yield in LRA evaluations. We conclude that LRA boosting approaches can affect the immune system differently and may work through different mechanisms. An LRA boosting approach that minimizes immune modulatory effects may be more appealing as an investigational intervention.

The variance in HIV-1 LRA responsiveness we document, across participants and between replicates, has implications for future studies. Small studies may be limited in their capacity to accurately quantify this variability, which increases with LRA potency and has been observed in other in vitro and ex vivo work (77–79). We speculate this variability may relate to heterogeneity in the HIV-1 reservoir or to variance in HIV-1 persistence measurements. This heterogeneity may be less about the absolute amount of provirus that may be found in a given PBMC aliquot and more about the capacity of a given set of proviruses, in their unique intracellular environments, to support virus transcription and its reactivation. Future work should first define the variance in cross-sectional replicate HIV-1 caRNA and poly(A) mRNA measurements from participant samples, in the absence of LRA. Assessments of LRA activity should prioritize larger participant sample sizes and more than 1 biological replicate of 5 million cells per participant. Ideally, the experimental conditions used to assess LRA efficacy could be standardized across the field, facilitating cross-study comparisons. We found that normalization of HIV-1 transcription to host gene transcription may introduce additional variability.

Despite HIV-1 reactivation that was superior to PMAi, multiply spliced HIV-1 transcription and virion production did not significantly increase with RMD in combination with low-dose bryostatin. Possible hypotheses that may explain these observations include that LRA boosting induces sequence-defective HIV-1 transcripts that cannot be spliced and/or translated, stimulates HIV-1 mRNA transcripts that remain trapped in the nucleus, or encounters a posttranscriptional block that limits splicing and virus translation (80–82). Blocks to HIV-1 splicing are increasingly recognized, and here we extend those findings to LRA boosting combinations (18–20, 83–85). PMAi did not induce supernatant viremia, after 24-hour incubation, in all participants; this heterogeneity has been reported (86). In CD4 samples from a subset of participants, however, we observed detectable supernatant viremia but not increases in the frequency of HIV-1 multiply spliced RNA (msRNA) with the LRA boosting combination of RMD and low-dose bryostatin. If an RNA splicing block limits LRA boosting–associated virion production, it may not be absolute. Also, virion production without significant HIV-1 msRNA increases may be due to virion production kinetics — i.e., msRNA may be translated and degraded as virions are produced — or may be due to assay technical differences between virion and msRNA quantifications. Future work should focus on the posttranscriptional mechanisms of HIV-1 RNA processing and interpatient differences in LRA responsiveness.

To standardize language, HIV-1 “latency reversal” may require more precise wording. Latency reversal can refer to the reactivation of virus unspliced or poly(A) mRNA transcription but can mean reversal of latency resulting in viral particle production. While some prior studies have identified HDACi-induced HIV-1 virion production ex vivo, this required HDACi exposures over 6–14 days, at times in combination with CD8 depletion or other LRA conditions that are more difficult to translate into investigational strategies (65, 75, 77, 87). Future work should clarify whether unspliced HIV-1 caRNA measurements are, in fact, the correct biomarker for LRA efficacy and whether other virus transcript or virion quantifications may provide more mechanistic and biological insight.

Our study has limitations. Prior and current injection opioid use limits the peripheral blood draw volumes we could obtain; this required us to prioritize the experiments with a given sample. The statistical significance of LRA boosting with AZD5582-containing regimens was not replicated in our HEAL cohort samples, and it is unclear if this relates to the variability in LRA response as sample size decreases or to unrecognized differences between our cohorts’ participants. We observed variance across clinical cohorts in the magnitude of LRA boosting responses to AZD5582 in combination with HDACi. While the sample sizes for these experiments differ (*n* = 36 vs. *n* = 9), and, in aggregate, the HIV-1 caRNA fold-change effects are clear, we cannot rule out that smaller sample sizes and/or unrecognized differences between our cohorts’ participants may affect the significance and the magnitude of latency reversal with some LRA boosting combinations. We did not specifically assess changes in HIV-1 proviral chromatin-associated histone acetylation. Additional mechanisms may explain how LRA boosting increases virus transcription; bryostatin may exert its HIV-1 latency reversal effects independently of PKC agonism (88).

Using HIV-1 LRA at submaximal doses and in combination with small molecules that do not increase virus transcription, ex vivo, expands understanding of how virus latency may be reversed. Implications of our study are that opioid use does not impede HIV-1 latency reversal, that increased sample sizes in LRA evaluations may mitigate variance in HIV-1 transcriptional responses, that some degree of HIV-1 transcriptional activity may be common during treated suppressed infection, and that more than 1 molecular event beyond histone acetylation will be required to reverse virus latency. Investigational LRA boosting regimens potentiate HIV-1 transcription and can be selected to minimize off-target immune-modulating effects. LRA boosting should be leveraged not to evaluate its clinical potential, but rather to probe molecular mechanisms and advance future effective LRA approaches. Follow-on studies should delineate the precise mechanisms required for efficient virus RNA processing and virion production during HIV-1 latency reversal.

## Methods

### Sex as a biological variable.

We studied blood samples obtained from male and female participants. Sex as a biological variable was assessed as an exploratory endpoint for LRA responsiveness.

### Study design and clinical cohorts.

The OPHION study is a prospective cohort study of PWH on suppressive ART recruited from Boston Medical Center (BMC), which serves one of the largest populations of PWH in Massachusetts, including a disproportionately large number of persons with opioid and substance use disorders. Eligible PWH were required to be taking ART and virologically suppressed (defined as undetectable plasma HIV-1 RNA or <20 copies/mL) for a minimum of 12 months prior to enrollment. Individuals were recruited into 1 of 5 cohorts: (a) active injection opioid use, defined as self-report on injecting opioids at least once per week, (b) methadone maintenance therapy for at least 3 months, (c) buprenorphine-naloxone therapy for at least 3 months, (d) prescription oral opioid use for at least 6 months, or (e) no opioid use within the past 1 year representing a control cohort. Eligibility into each of the 5 cohorts was confirmed with a urine toxicology performed at the BMC central laboratory, which includes evaluation for urine drug metabolites including opiates and nonopioid substances as well as an expanded opioid panel, which included fentanyl or norfentanyl metabolites. Exclusionary criteria included ongoing immunosuppression and pregnancy; patients who reported injecting nonopioid substances were also not eligible. Once consented and enrolled, each participant underwent blood collection for same-day PBMC extraction as well as urine collection for gabapentin and benzodiazepine metabolites. Participants also completed an extensive survey on their current and prior substance use (including alcohol and tobacco use) based on the addiction severity index (89) and Texas Christian University drug screen (90). HIV and other clinical information, including ART treatment history, duration of virologic suppression, and comorbidity status including hepatitis C and B, were abstracted from the medical record.

The HEAL cohort is a longitudinal study of PWH. Inclusion criteria for HEAL participants in this study were 1) ART-treated participants with HIV-1 who were virologically suppressed, defined as plasma HIV-1 RNA less than assay (most commonly less than 20 copies/mL, without blips) for >1 year, who 2) had sufficient available amounts of cryopreserved PBMCs for the proposed analyses. Experimenters were masked to all clinical data and to OPHION subgroup assignments.

### Quantification of HIV-1 persistence markers.

Genomic DNA and total RNA were isolated from approximately 5 × 10^6^ PBMCs or tCD4 (AllPrep DNA/RNA Kit, QIAGEN). Total HIV-1 DNA and unspliced caRNA levels were quantified in triplicate by real-time PCR as previously described, modified to include Taqman Universal Mastermix (DNA) or Taqman Fast Virus 1-Step Mastermix (RNA) (91). HIV-1 quantifications used forward primer 5′-TACTGACGCTCTCGCACC-3′, reverse primer 5′-TCTCGACGCAGGACTCG-3′, and probe 5′ FAM-CTCTCTCCTTCTAGCCTC-MGB 3′ (Thermo Fisher Scientific). DNA cycling conditions in a total reaction volume of 25 μL were 95°C for 15 minutes followed by 40 cycles of 95°C for 15 seconds and 60°C for 1 minute. RNA cycling conditions in a total reaction volume of 20 μL were 55°C for 15 minutes, 95°C for 20 seconds, followed by 40 cycles of 95°C for 3 seconds and 60°C for 30 seconds. Approximately 500 ng of genomic DNA was assessed per well. The limits of quantification for total HIV-1 DNA and unspliced caRNA were 1 and 3 copies per reaction, respectively. Limits of detection were calculated per time point and participant sample, considering the average number of copies detected across triplicate measurements. Cell input numbers were quantified by human genome equivalents of CCR5 DNA using forward primer 5′-ATGATTCCTGGGAGAGACGC-3′, reverse primer 5′-AGCCAGGACGGTCACCTT-3′, and probe 5′ FAM-CTCTCTCCTTCTAGCCTC-MGB 3′ (Thermo Fisher Scientific) (91).

### Intact proviral DNA assay.

HIV-1 DNA from isolated PBMC DNA was measured using the intact proviral DNA assay as previously described with minor modifications (92). Two multiplexed droplet digital PCR assays (Bio-Rad QX200) were performed per sample targeting conserved HIV-1 sequences and, to normalize data, a cell reference gene. Master mixes were prepared using 2× ddPCR Supermix for probes. The HIV-1–specific reaction targeted the *Psi* packaging signal (forward 5′-CAGGACTCGGCTTGCTGAAG-3′; Reverse 5′-GCACCCATCTCTCTCCTTCTAGC-3′; probe 5′-TTTTGGCGTACTCACCAGT-3′, FAM, MGB) and *env* (forward: 5′-AGTGGTGCAGAGAGAAAAAAGAGC-3′; reverse: 5′-GTCTGGCCTGTACCGTCAGC-3′; probe intact: 5′-CCTTGGGTTCTTGGGA-3′, VIC/HEX, MGB) sequences with 700 ng DNA per well in duplicate. An additional probe without a fluorophore targeting hypermutated *env* sequence (probe hypermutated: 5′-CCTTAGGTTCTTAGGAGC-3′, unlabeled, MGB) was used to exclude defective gene quantification. To estimate the number of cells per reaction and correct for sheared DNA, separate reactions targeting 2 regions of *RPP30*, RPP30-1 (forward: 5′-GATTTGGACCTGCGAGCG-3′; reverse: 5′-GCGGCTGTCTCCACAAGT-3′; probe: 5′-CTGACCTGAAGGCTCT-3′, VIC/HEX, MGB) and RPP30-2 (forward: 5′-GACACAATGTTTGGTACATGGTTAA-3′; reverse: 5′-CTTTGCTTTGTATGTTGGCAGAAA-3′; probe: 5′-CCATCTCACCAATCATTCTCCTTCCTTC-3′, VIC/HEX), with similar nucleotide distance between targets compared to *Psi* and *env* were prepared with 70 ng DNA per well. Droplets were generated and subjected to PCR: 95°C for 10 minutes, followed by 45 cycles of 94°C for 30 seconds, 59°C for 1 minute, and ending with 98°C for 10 minutes before holding at 4°C. Data from PCR products were collected and analyzed using QuantaSoft Data Analysis Software. Droplets containing a double-positive signal (*Psi*^+^*env*^+^) were considered intact, while single-positive droplets represented defective provirus containing 5′ deletions (*Psi*^–^*env*^+^) or 3′ deletions/hypermutations (*Psi*^+^*env*^–^). Relative DNA shearing was calculated using the ratio of single-positive RPP30 droplets compared with the double-positive population and was used to estimate the number of intact proviruses. The number of diploid cells in each reaction was determined by dividing the copies of RPP30 in half and correcting for the dilution factor for the HIV-1 reaction. Intact, defective, and total HIV-1 DNA were normalized to the number of cells and reported as copies/million PBMCs.

### msHIV-1 transcripts.

To measure the frequency of total CD4^+^ T cells with inducible msHIV-1 RNA, we performed the TILDA, as previously described, with 2 modifications (51). First, to reduce the 95% CIs around our frequency estimates, we used 36 × 10^6^ cells/mL as our first dilution, rather than the 18 × 10^6^ cells/mL used in Procopio et al. Second, to harmonize our PMAi concentrations with LRA experiments, we reduced the PMA concentration from its original 100 ng/mL to 50 ng/mL in our TILDAs. The duration of virologic suppression in our HEAL participants was similar to previously reported participants (51).

### Reference gene quantification.

Extracted PBMC RNA was used to quantify transcription levels of 3 host genes, IPO8, TBP, and UBE2D2, typically immediately after the loading of HIV-1 caRNA qPCR plates was completed. IPO8 quantifications used forward primer 5′-CCTTTGTACAACAGAAGGCAC-3′, reverse primer 5′-TGCACGTCTCAGGTTTTTGC-3′, and probe 5′ FAM-TCCGCATAAATCCATTGATTCTGC-MGB 3′ (Thermo Fisher Scientific); TBP quantifications used forward primer 5′-CAGTGAATCTTGGTTGTAAACTTGA-3′, reverse primer 5′-TCGTGGCTCTCTTATCCTCAT-3′, and probe 5′ FAM-CGCAGCAAACCGCTTGGGATTAT-MGB 3′ (Thermo Fisher Scientific); and UBE2D2 quantifications used forward primer 5′-GTACTCTTGTCCATCTGTTCTCTG-3′, reverse primer 5′-CCATTCCCGAGCTATTCTGTT-3′, and probe 5′ VIC-CCGAGCAATCTCAGGCACTAAAGGA-MGB 3′ (Thermo Fisher Scientific). To generate IPO8 standards, we cloned a fragment corresponding to nucleotides 3636–3707 of IPO8 mRNA (Sequence ID NM_006390.4) into a pCR4-TOPO vector (Thermo Fisher Scientific, catalog K458001). To generate TBP standards, a fragment corresponding to nucleotides 649–1037 of TBP mRNA (Sequence ID NM_003194) was cloned into a pCR4-TOPO vector. To generate UBE2D2 standards, a fragment corresponding to nucleotides 729–1164 of UBE2D2 mRNA (Sequence ID NM_181838.2) was cloned into a pCR4-TOPO vector. Reference gene RNA was synthesized using T3 Megascript Kit (Thermo Fisher Scientific catalog AM1333 and cleaned (RNeasy MinElute Cleanup Kit, QIAGEN, catalog 74204) before serially diluting across a range of 10^6^–10^3^ copies per well. After TBP and UBE2D2 standards were individually validated, TBP (T) and UBE2D2 (U) RNA were diluted separately and aliquoted together as 1 set of multiplex standards. T and U standards were validated to demonstrate overlap of individual and multiplexed standard curves. The assay was optimized to run all host gene standards and samples on one 96-well reaction plate in a total reaction volume of 20 μL using cycling conditions of 55°C for 5 minutes, 95°C for 20 seconds, followed by 40 cycles of 95°C for 3 seconds and 56°C for 30 seconds. Reference gene standards and samples were run in duplicate. IPO8, TBP, and UBE2D2 RNA copies per million PBMCs were calculated by dividing copies per total sample by number of cells per sample, determined by CCR5 quantification, and normalized to 1 × 10^6^ cells. Fold-changes were calculated by dividing the RNA copy number obtained per million PBMCs for each LRA condition by the 0.2% DMSO control condition’s reference gene RNA copies/million PBMCs.

### HIV-1 latency reversal.

OPHION and HEAL participant PBMCs were isolated from large-volume (90–180 cc) peripheral blood or leukapheresis collections by density centrifugation and cryopreserved. Cryopreserved PBMCs were thawed, pelleted, and transferred to RPMI media supplemented with 10% fetal bovine serum, 2 μM raltegravir, and 2 μM tenofovir (R10 + TDF/RAL) at a concentration of 1 × 10^6^ cells/mL. Cells were rested in a humidified CO_2_ incubator at 37°C for 3 hours. After 3 hours, cells were counted and assessed for viability. For CD4 experiments, PBMCs were pelleted, resuspended in EasySep Buffer (STEMCELL Technologies, catalog 20144), and isolated with EasySep Human CD4^+^ T Cell Enrichment Kit (STEMCELL Technologies catalog 19052) per the manufacturer’s instructions. A total of 5 × 10^6^ PBMCs, or tCD4, were aliquoted into 5 mL of fresh R10 + RAL/TDF in 6-well sterile cell culture plates. Cells were incubated in the presence or absence of the following LRA conditions: 0.2% DMSO, 1:1 αCD3/αCD28 beads (Life Technologies, catalog 11131D) to cells with 30 U/mL IL-2 (R&D Systems, Bio-Techne catalog 202-IL-500), RMD 20 nM (MilliporeSigma, catalog SML1175), PNB 30 nM (MilliporeSigma, catalog SML3060), bryostatin-1 1 nM (MilliporeSigma, catalog B7431), AZD5582 100 nM (ChemieTek, catalog CT-A5582), RMD 20 nM plus bryostatin-1 1 nM, PNB 30 nM plus bryostatin-1 1 nM 100 nM, RMD 20 nM plus AZD5582 100 nM, PNB 30 nM plus AZD5582 100 nM, and PMA 50 ng/mL (MilliporeSigma, catalog P1585) in combination with ionomycin 1 μM (MilliporeSigma, catalog I9657). To provide a known positive control for subsequent immunology assessments, αCD3/αCD28 beads were used with OPHION samples. Cultures were incubated at 37°C for 24 hours. After 24 hours, cells were prepared for nucleic acid extractions.

### Comparison of qPCR assays after reactivation.

CD4^+^ T cells were isolated from cryopreserved HEAL participant PBMCs. tCD4 were isolated using a CD3^+^CD4^+^CD8^–^ isolation kit (STEMCELL Technologies catalog 17952), and rCD4 were isolated using a CD3^+^CD4^+^CD8^–^CD25^–^CD69^–^HLA-DR^–^ isolation kit (STEMCELL Technologies catalog 17962). Cells were pelleted and transferred to RPMI medium supplemented with 10% fetal bovine serum, 2 μM raltegravir, and 2 μM tenofovir (R10 + TDF/RAL) at a concentration of 5 × 10^6^ cells/mL. Cells were rested in a humidified CO_2_ incubator at 37°C for 3 hours and then counted and assessed for viability. A total of 5 × 10^6^ tCD4 or rCD4 were aliquoted into either 1 mL or 5 mL of fresh R10 + RAL/TDF in a 24-well sterile cell culture plate or 6-well sterile cell culture plate, respectively. Cells were incubated in either 0.2% DMSO or PMA 50 ng/mL (MilliporeSigma, catalog P1585) in combination with ionomycin 1 μM (MilliporeSigma, catalog I9657). Cultures were incubated at 37°C for 24 hours. After 24 hours, cells were prepared for nucleic acid extractions. RNA was eluted in a total volume of 65 μL and DNA eluted in a volume of 100 μL. For HIV-1 unspliced caRNA quantifications, 10 μL of RNA were assessed in triplicate using primers and probe that bind the HIV-1 *gag* region (91). To calculate copies of HIV-1 caRNA per million cells, copy numbers were averaged and cell counts normalized to a CCR5 DNA reference. For poly(A) HIV-1 mRNA quantifications, 10 μL (approximately 1 million cell equivalents) of RNA were run in triplicate using primers and probe targeting the nef to poly(A) region of HIV-1 mRNA as previously described (23). Our HIV-1 unspliced and poly(A) caRNA amplifications are both 1-step qPCR assays that use an HIV-specific primer to synthesize cDNA. Standards were generated as previously described (82). For poly(A) HIV-1 mRNA quantifications, copies per million cell equivalents were calculated by initial cell count prior to reactivation. Both assays used Taqman Fast Virus 1-Step Mastermix in a total reaction volume of 20 μL and cycling conditions of 55°C for 15 minutes, 95°C for 20 seconds, followed by 40 cycles of 95°C for 3 seconds and 60°C for 30 seconds. Fold-change was calculated by dividing copies per million cells in the PMAi condition by copies per million cells in the 0.2% DMSO no-drug control condition for each sample.

### AIM assay.

AIM assays were performed as previously described (49). Briefly, cryopreserved PBMCs were thawed, washed, resuspended in R10, and rested for 3 hours at 37°C. Following the 3-hour rest interval, the appropriate number of cells were transferred to a 48-well plate and subsequently treated with CD40 blocking antibody (Miltenyi Biotec, catalog 130-094-133) for a final concentration of 0.5 μg/mL for 15 minutes at 37°C. Cells were incubated in the presence or absence of our 10-LRA panel, as previously described. After an 18-hour incubation, cells were harvested and stained for 50 minutes at 4°C with the surface staining mAbs (see Supplemental Methods for RRIDs): PD-L1-PE/Cy7 (BioLegend, catalog 329717), CD40L-PE (BD Biosciences [BD], catalog 561720), OX40-APC (BD catalog 563473), CD69-BV650 (BioLegend catalog 310933), CD3-BV605 (BioLegend catalog 317322), CD4-BV421 (BD catalog 562424), CD8-PerCp-Cy5.5 (BD catalog 560662), CD25- BUV395 (BD catalog 564034), and LIVE/DEAD Near-IR stain (Thermo Fisher Scientific catalog L34975). Then they were washed, fixed and permeabilized (BD cytofix fixation buffer, catalog 554655), and stained for 30 minutes at 4°C with intracellular mAb Acetyl-histone H3-Alexa Fluor 488 (Cell Signaling Technology, catalog 9683S) in 1× perm/wash buffer (BD, catalog 554723). The cells were washed and fixed (BD cytofix fixation buffer, catalog 554655) prior to flow cytometry analysis.

### Intracellular cytokine staining.

Cryopreserved PBMCs were thawed, washed, resuspended in RPMI + 10% FBS (R10), and rested for 3 hours at 37°C. Following the 3-hour rest interval, the appropriate number of cells were transferred to a 48-well plate and incubated for 2 hours with our panel of 10 LRAs. Following this 2-hour incubation period, GolgiPlug (BD, catalog 555029) and GolgiStop (BD, catalog 554724) were added to each condition at a concentration of 1:1,000 and 6:10,000, respectively, then incubated at 37°C for an additional 16 hours. Cells were harvested, pelleted, resuspended, stained for 20 minutes at 4°C with surface staining mAbs (CD3-BV605, CD4-BV421, CD8-PerCp-Cy5.5 and LIVE/DEAD Near-IR stain), fixed and permeabilized as described above, and then stained for 30 minutes at 4°C with the intracellular mAbs TNF-α–PE/Dazzle-594 (BioLegend catalog 502946), IL-2–PE/Cy7 (BD catalog 560707), and IFN-γ–BV510 (BioLegend catalog 502544). PBMCs were washed and fixed in BD cytofix fixation buffer (catalog 554655) prior to flow cytometry analysis, as described above.

### Flow cytometry.

Cells were acquired on a BD LSRFortessa using FACSDiva. Analysis was performed using FlowJo software version 10.6.2 (TreeStar). Representative gating strategies can be found in Supplemental Figure 3.

### Statistics.

Statistical analyses were performed using GraphPad Prism 9. Means, medians, 95% CIs, and SEM were calculated. The prespecified primary outcome of OPHION was a comparison of log_10_ PBMC HIV-1 caRNA levels between participants who use and do not use opioids. The prespecified outcome for OPHION latency reversal experiments was the difference in HIV-1 caRNA levels between LRA treatment groups and across opioid use groups. To assess for changes in HIV-1 caRNA, we performed Kruskal-Wallis testing of all participants together for each of the LRAs with Dunn’s multiple-comparison test, comparing the mean ranks of LRA conditions. Similar approaches were taken with a series of exploratory analyses with OPHION participant samples that assessed histone acetylation, surface activation, and intracellular cytokine production, and with the exploratory statistical analyses performed on experiments with HEAL participant samples, unless otherwise specifically stated in the text.

### Study approval.

The IRBs at Boston Medical Center and Brigham and Women’s Hospital (Mass General Brigham Human Research Committee) approved the study protocols. Written informed consent was obtained from all participants.

### Data availability.

The data supporting the findings of this study are available within the Supporting Data Values file or from the corresponding author upon request.

## Author contributions

AA, JT, KC, NL, and AT conceived the study. AA, JT, KC, NL, and AT developed methodology. TL, JB, AA, AO, KC, HC, HJ, and KC investigated. TL, JB, AA, AO, KC, and AT visualized data. NL and AT acquired funding. AA, SR, NL, and AT were project administrators. AA, NL, and AT were supervisors. TL, JB, AA, AO, NL, and AT wrote the original draft. TL, AA, NL, and AT reviewed and edited the manuscript.

## Supplementary Material

Supplemental data

Supporting data values

## Figures and Tables

**Table 1:**
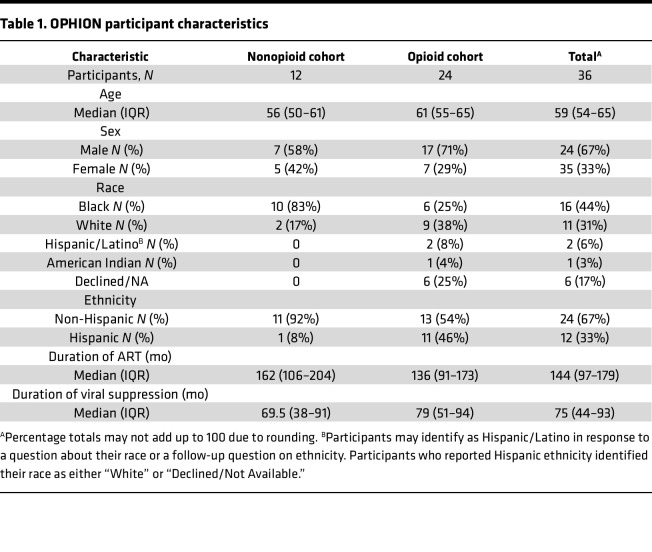
OPHION participant characteristics

## Version 1 10/29/2024

In-Press Preview

## Version 2 11/22/2024

Electronic publication
