# Supplementary material for: HIV-1 latency reversal agent boosting is not limited by opioid use
Source: JCI Insight. 2024 Nov 22;9(22):e185480. doi: 10.1172/jci.insight.185480 (PMC11601940; doi:10.1172/jci.insight.185480)
Supplement: Supplemental data [file jciinsight-9-185480-s261.pdf]

Supplementary Methods

Activation Induced Marker (AIM) assay

Surface staining mAbs used were as follows:

**Antibodies**	**Source**	**Identifier**
CD40 Antibody, anti-human	Miltenyi Biotec	Cat#130-094-133; RRID:AB_10839704
PD-L1-PE/Cy7	Biolegend	Cat#329717; RRID:AB_2561687
CD40L-PE	BD Biosciences	Cat#561720; RRID:AB_10924597
OX40-APC	BD Biosciences	Cat#563473; RRID:AB_2738230
CD69-BV650	Biolegend	Cat#310933; RRID:AB_2563158
CD3-BV605	Biolegend	Cat#317322; RRID:AB_11126166
CD4-BV421	BD Biosciences	Cat#562424; RRID:AB_11154417
CD8-PerCp-Cy5.5	BD Biosciences	Cat#560662; RRID:AB_1727513
CD25- BUV395	BD Biosciences	Cat#564034; RRID:AB_2738556
LIVE/DEAD Near-IR stain	Thermo Fisher Scientific	Cat#L34975
Acetyl-histone H3-Alexa Fluor 488	Cell Signaling Technology	Cat#9683S; RRID: AB_2661899
TNF-α- PE/Dazzle-594	Biolegend	Cat#502946; RRID:AB_2564172
IL-2- PE/Cy7	BD Biosciences	Cat#560707; RRID:AB_1727542
IFN-γ- BV510	Biolegend	Cat#502544; RRID:AB_2561464

Supplementary Figures and Tables

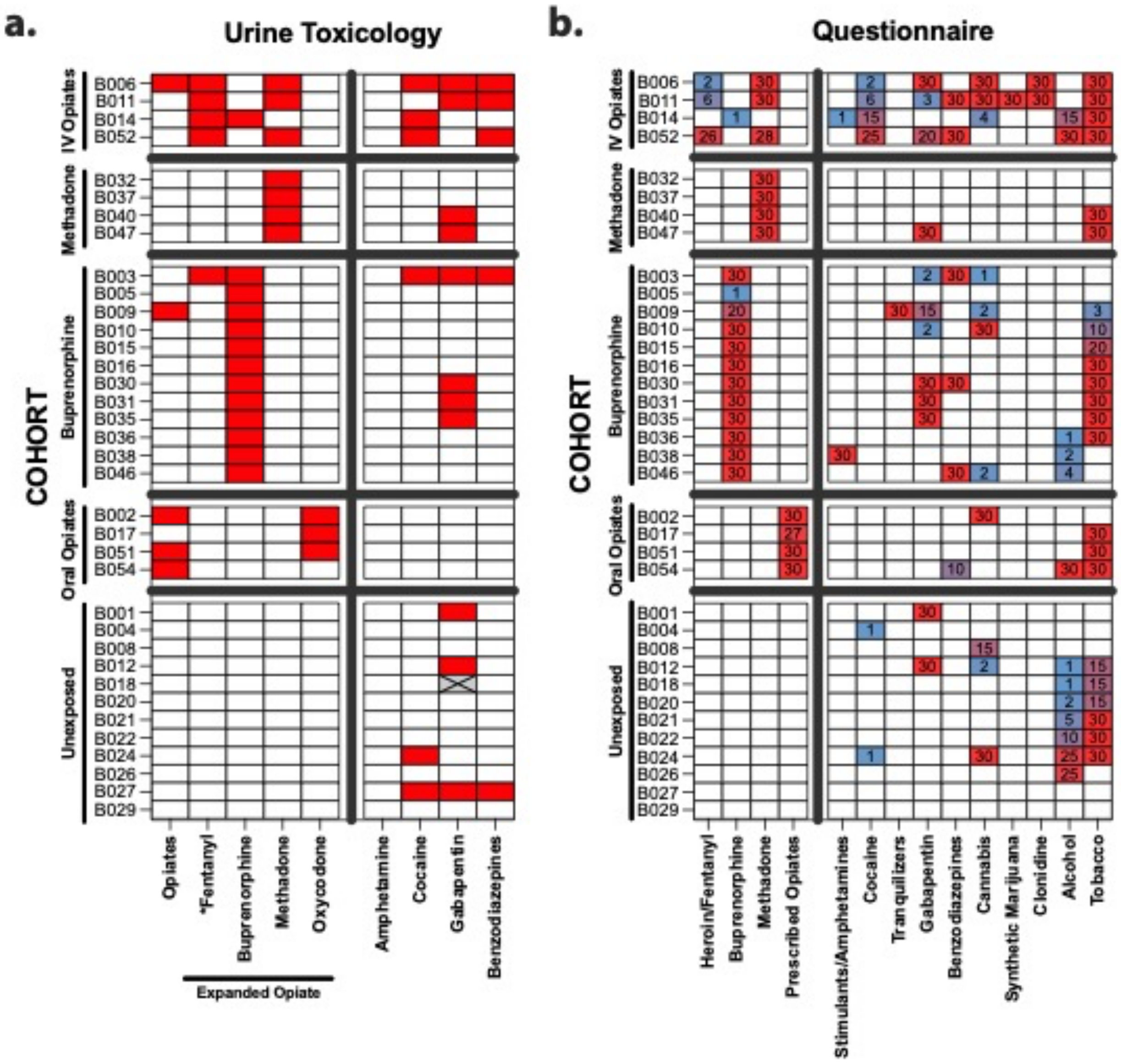

Supplementary Figure 1. Substance use in the OPHION cohort. We assessed substance use by (a) clinical urine toxicology testing and (b) self-reported substance use questionnaires. Substance use results are separated by cohort groups by participant ID. Red squares indicate the presence of that substance in a urine sample; unavailable data were marked with “X” in a gray box. An expanded opiate panel was performed to assess for the presence of different opiate formulations.

Color squares in panel b reflect any reported use by participants in the last 30 days. The numbers inside a given box indicate the number of days used this substance was reported to be used in the last 30 days. Gradient of color from blue to red indicate increasing report of use whereas white indicates no use. Concomitant use of alcohol, tobacco, marijuana, and gabapentin in the OPHION cohort was common. *all fentanyl measures were confirmed by follow-on quantification. Participants in the buprenorphine and methadone groups reported daily use of the respective medication in the 30 days prior, whereas participants taking opioids for chronic pain took opioids for 27-30 days in the 30 days preceding their enrollment in OPHION. 75% (N=3/4) of active injection users had urine toxicology screens positive for cocaine corresponding with self-report; all three reported smoking cocaine in the prior 30 days to specimen collection. One of twelve participants in the methadone use group and 2/12 in the non-opiate use group also has urine toxicology screens positive for cocaine. All active injection opioid users reported using multiple drugs on the same day in the last 30 days compared to 1/12 participants in non-opioid use group and 10/20 participants in methadone, buprenorphine and prescription opiate use groups combined. N=3/4 active injection opioid users reported smoking marijuana in the last 30 days. 6 and 8 participants about the 12 participants in opioid use groups had urine toxicology screens positive for benzodiazepines and gabapentin; all but 1 in both cases reported that this substance was prescribed. No participant had urine toxicology screens positive for amphetamines or barbiturates. All participants injecting opioids used tobacco in the preceding 30 days and, overall, 23 of 36 OPHION participants used tobacco within the month prior to their blood collection. 25% (N=6/24) of opioid use participants consumed alcohol in last 30 days compared to 58% (7/12) in the control group.

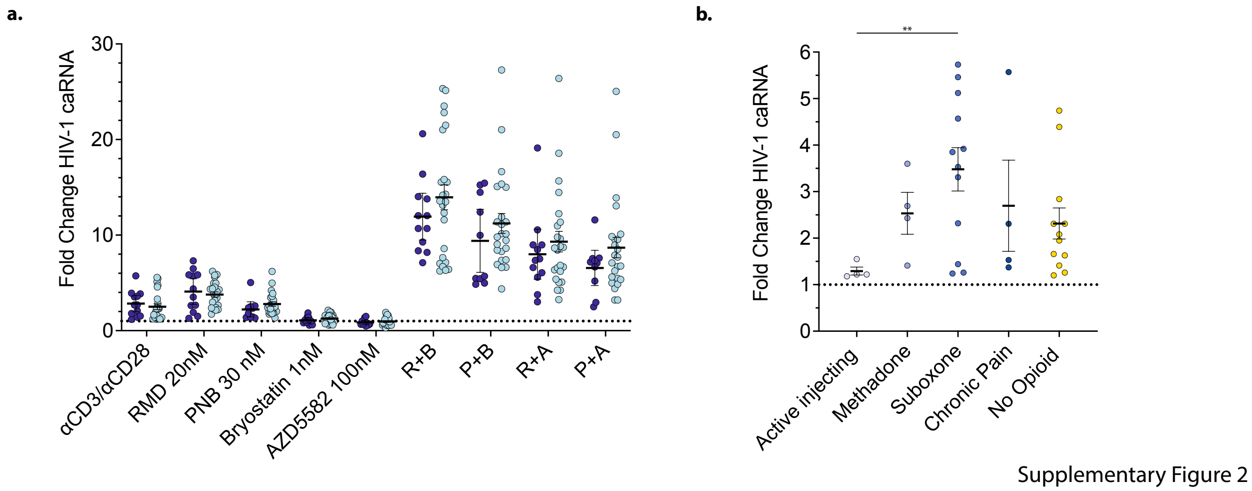

Supplementary Figure 2. HIV-1 LRA boosting response in the OPHION cohort by subgroup analysis. (a) HIV-1 fold-increase in unspliced RNA transcription plotted as a function of self-reported Hispanic (dark purple circles) or non-Hispanic (light blue circles) ethnicity. (b) Opioid-use subgroup analysis highlighting LRA response to αCD3/αCD28 beads from Main Fig 1a, as a function of opioid use subgroup. Means and SEM are shown. Dotted horizontal line denotes a fold-change of 1. ** $p<0.01$ for LRA combinations, corrected for multiple comparisons. αCD3/αCD28, anti-CD3 anti-CD28 superparamagnetic beads; RMD, romidepsin; PNB, panobinostat; R, RMD 20nM; P, PNB 30nM; B, bryostatin 1nM; A, AZD5582 100nM.

Supplementary Figure 3

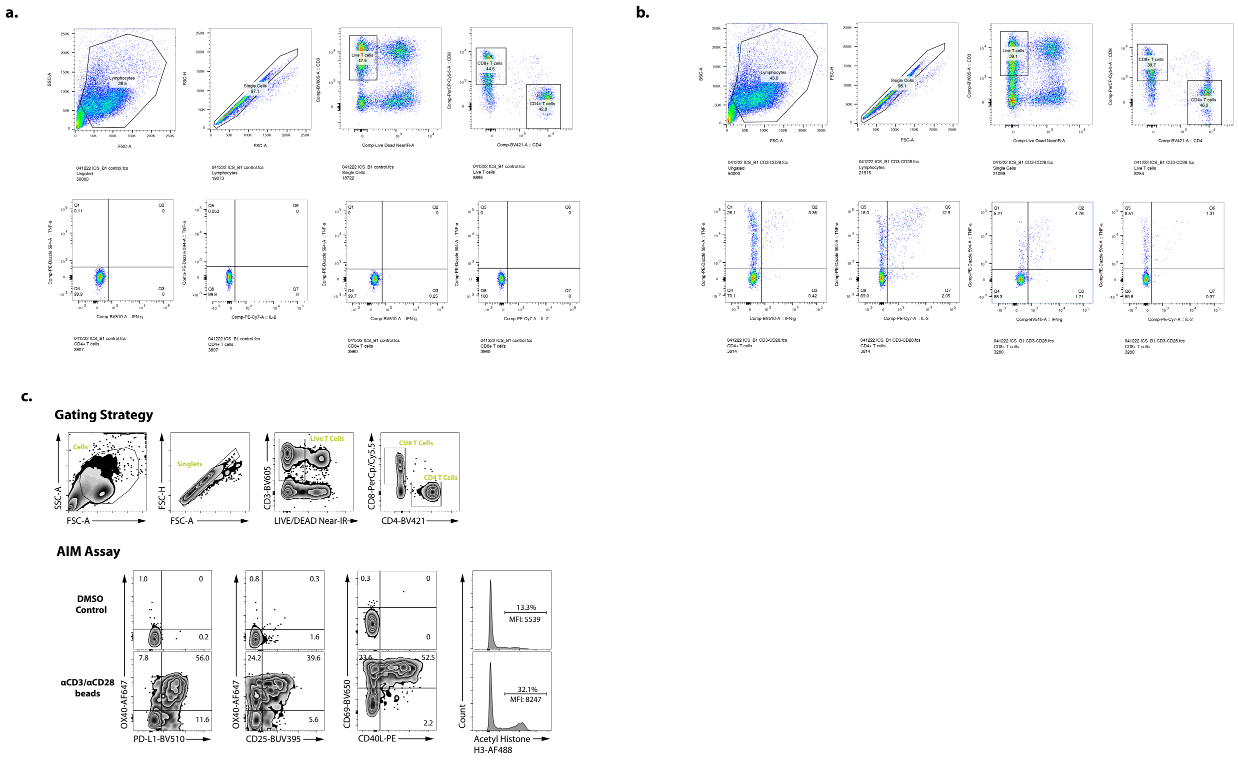

Supplementary Figure 3. Flow cytometry gating strategies. Representative intracellular cytokine staining plots for (a) control and (b) αCD3/αCD28 bead-exposed PBMC are shown. (c) Gating strategy for the three AIM assays. A representative sample exposed to TCR agonism is shown.

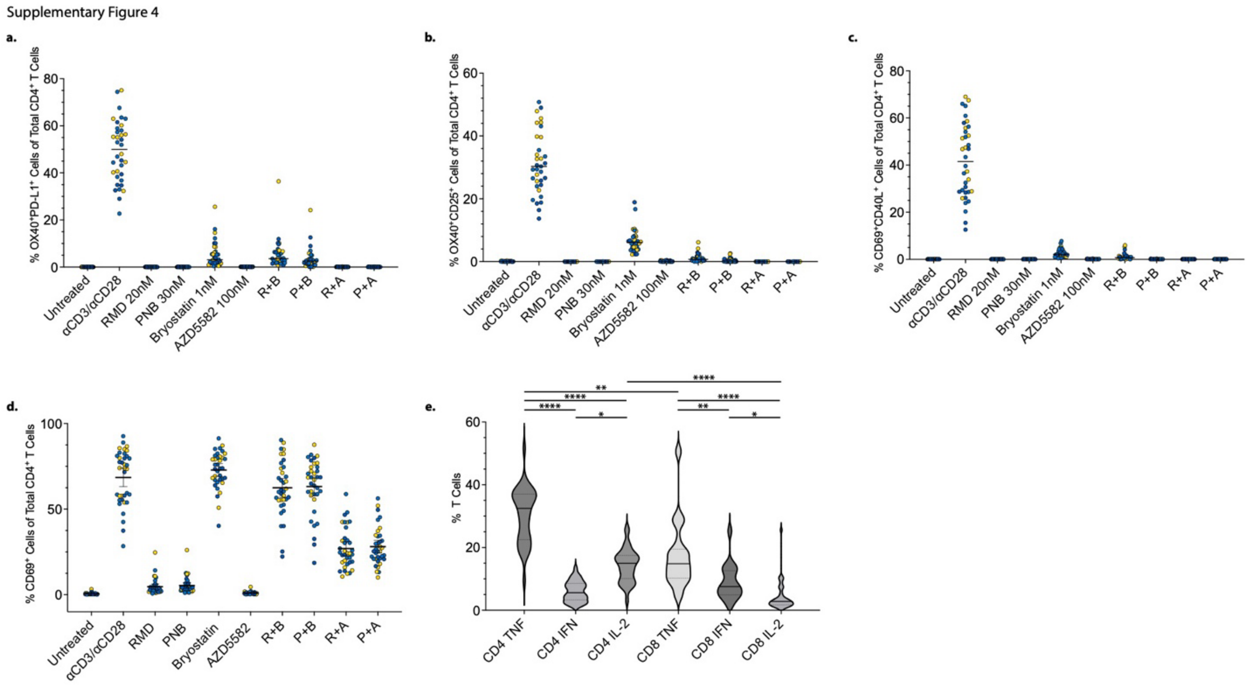

Supplementary Figure 4. LRA-induced immune activation as a function of opioid use. Results obtained with the (a) OX40/PD-L1, (b) OX40/CD25, and (c) CD69/CD40L AIM assays are reported for all ten conditions in the LRA panel, where samples from participants with (blue circles) and without (yellow circles) opioid use are shown. Data are represented as means ± 95% CIs. For the OX40/PL-L1 assay, the highest measured levels in OX40/PDL1 assay with bryostatin 1nM, R+B, and P+B are the same non-opioid-using participant. (d) Individual data points for $CD69^{+}$ live total CD4+ T cells, as a function of opioid use. (e) Comparative hierarchies of intracellular cytokine production in $CD4^{+}$ and $CD8^{+}$ T cells exposed to TCR agonism (αCD3/αCD28 beads). Data displayed in these panels are identical to the data values shown in Main Fig. 3. *p<0.05, ** p<0.01, **** p<0.0001, corrected for multiple comparisons. TNF, tumor necrosis factor alpha; IFN, interferon gamma; IL-2, interleukin-2; CD4, $CD4^{+}$ T cells; CD8, $CD8^{+}$ T cells.

Supplementary Figure 5

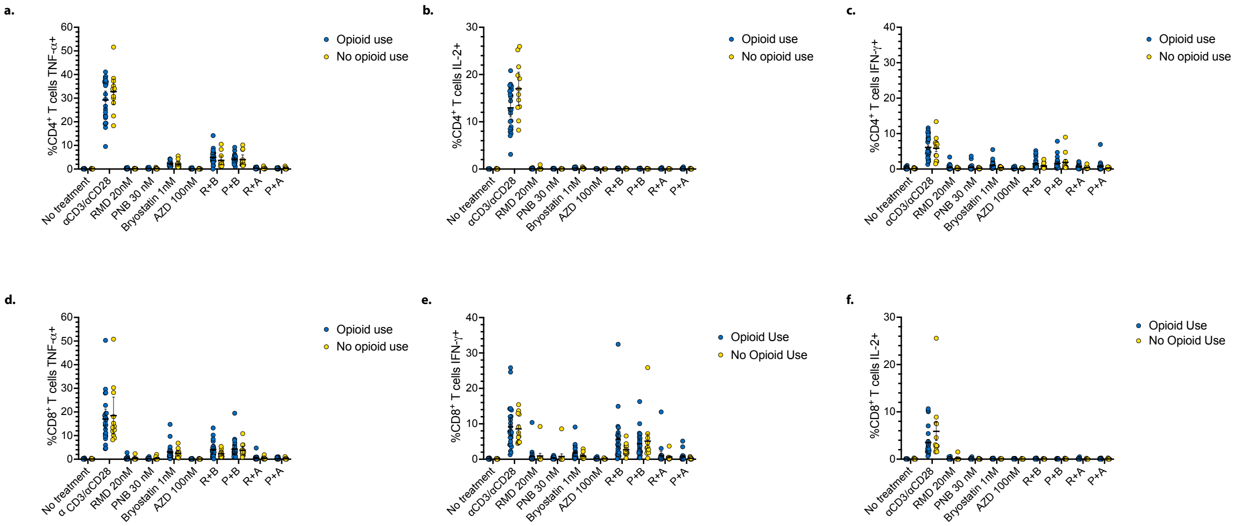

Supplementary Figure 5. Intracellular cytokine production, as a function of opioid use. Using the same data displayed in Main Fig. 3, here we show individual data points, labelled as samples from participants with (blue circles) and without (yellow circles) opioid use. CD4+ T cell production of (a) TNF-α, (b) IL-2, and (c) IFN-γ. CD8+ T cell production of (d) TNF-α, (e) IFN-γ, (f) IL-2. No significant differences in cytokine production were observed by opioid use.

Supplementary Figure 6

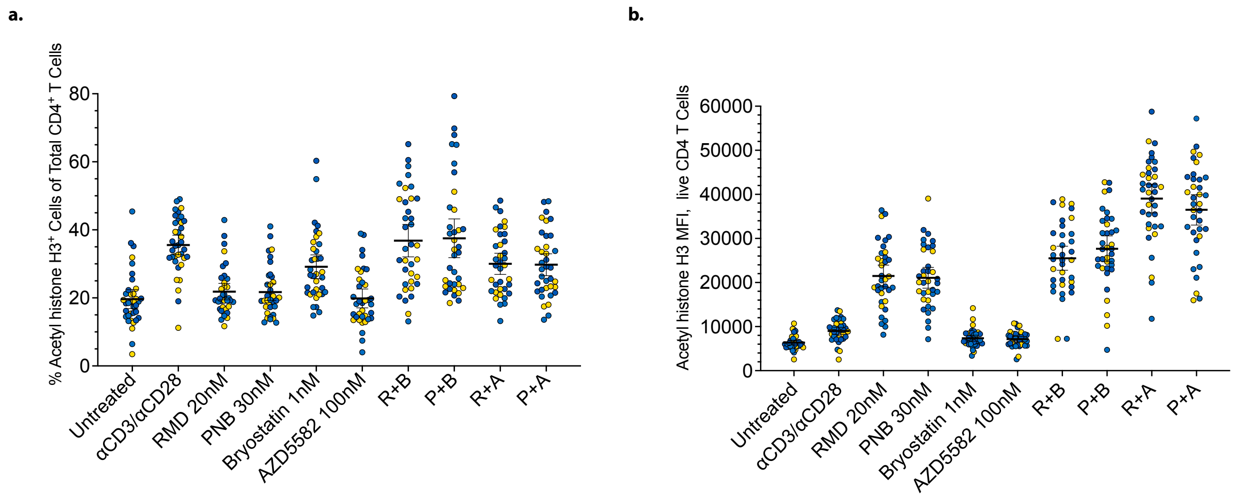

Supplementary Figure 6. Histone acetylation effects during LRA boosting, as a function of opioid use. Using the same data displayed in Main Fig. 4, here we show individual data points, labelled as samples from participants with (blue circles) and without (yellow circles) opioid use. (a) The proportion of live total CD4$^+$ T cells with acetylated histone H3, assessed by flow cytometry, (b) Mean fluorescence intensity (MFI) of acetylated histone H3 per live total CD4$^+$ T cells.

Supplementary Figure 7

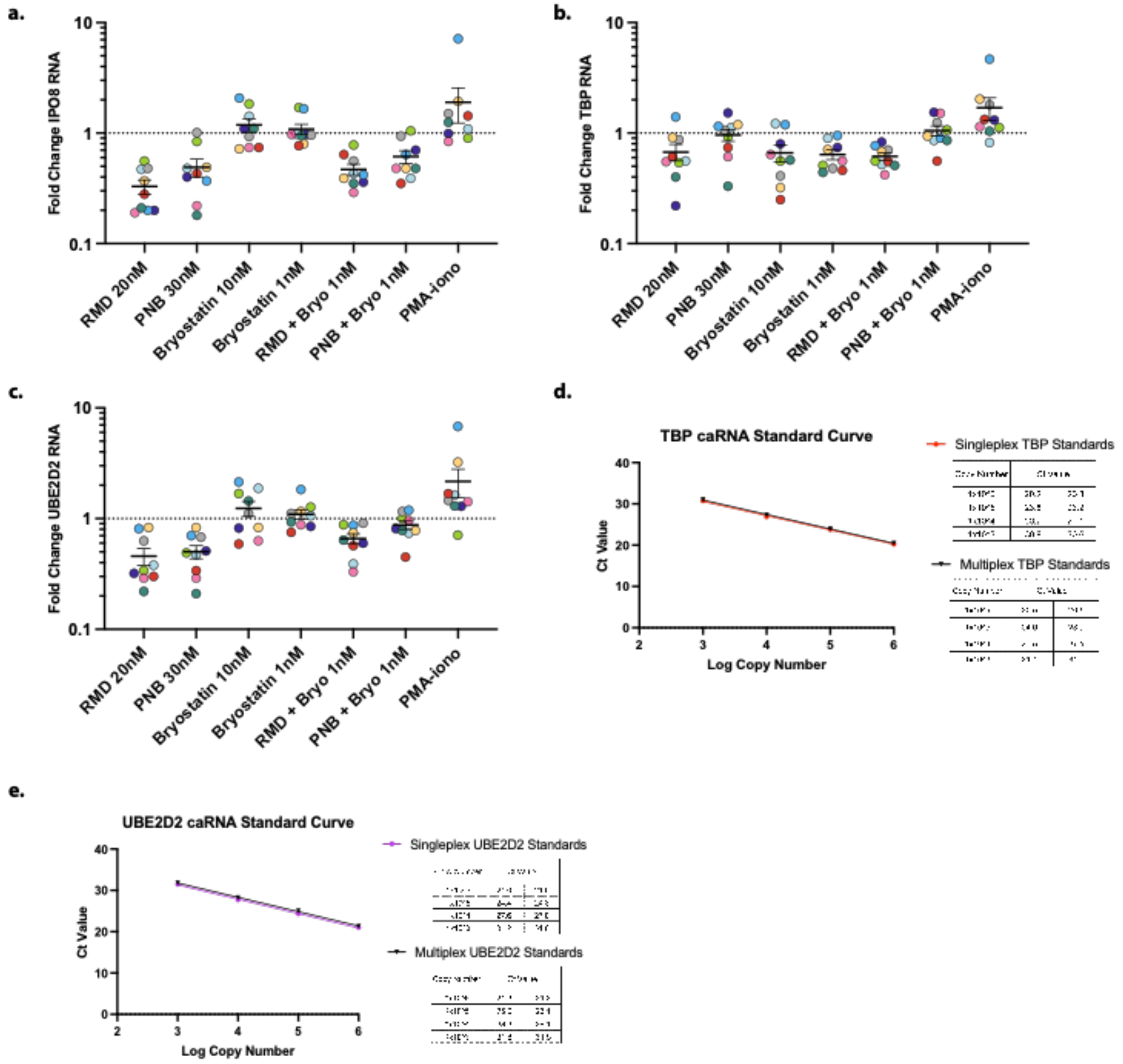

Supplementary Figure 7. The effects of LRA boosting on human reference gene transcription. Using leukapheresis samples from HEAL participants (n=9), we assessed and compared fold changes in transcription of three host genes: (a) IPO8, (b) TBP, and (c) UBE2D2. Reference gene multiplexing was validated for (d) TBP and (e) UBE2D2 quantifications. The rationale to study these three reference genes is as follows. IPO8 transcription is used as an internal control by the AIDS Clinical Trials Group's Virology Specialty Laboratories to measure RNA integrity in a dichotomous way. IPO8 Ct values >27 are used to suggest degradation of cellular samples in

storage, and samples with IPO8 Ct >27 do not report HIV-1 RNA values. Recent work by Nancie Archin at the UNC HIV Cure Center identified TBP and UBE2D2 as two of the more stable host genes during LRA exposures, which included PMA, AZD5582, and HDACi, among a panel of eight LRA.

Supplementary Figure 8

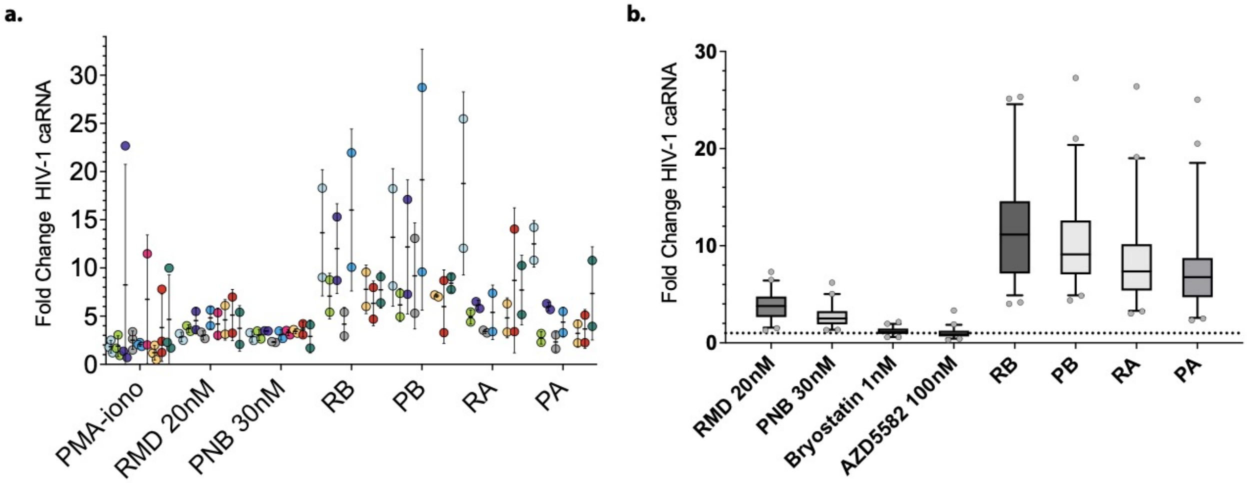

Supplementary Figure 8. Variance in LRA response. (a) Magnitude of HIV-1 caRNA transcription fold-changes in biological replicate LRA experiments performed with HEAL leukapheresis samples, using the same data as shown in Main Fig 5a-c, (b) Box and whiskers plot displaying the data of Fig. 5g as the mean (solid black horizontal line), inter-quartile range (25th – 75th percentiles denoted as the borders of the vertical rectangle), and 5th-95th percentiles (whiskers, error bars). Values below and above the whiskers are shown as individual data points. PMA-iono, PMA with ionomycin; RMD, romidepsin; PNB, panobinostat; R, RMD 20nM; P, PNB 30nM; B, bryostatin 1nM; A, AZD5582 100nM.

Supplementary Table 1 – Breakdown of OPHION cohort into opioid use subgroups

Supplementary Table 1. OPHION Opioid Use Sub-Group Characteristics

Characteristic	Active injection	Methadone	Suboxone	Chronic Pain	No opioid	**Total***
Participant, N	4	4	12	4	12	36
Age (years)						
Median (IQR)†	56 (46-61)	59 (55-65)	61 (57-67)	61 (57-67)	64 (59-65)	59 (54-65)
Sex						
Male N (%)	4 (100%)	2 (50%)	9 (75%)	2 (50%)	7 (58%)	24 (67%)
Race						
Black N (%)	1 (25%)	1 (25%)	3 (25%)	1 (25%)	9 (75%)	15 (42%)
White N (%)	1 (25%)	2 (50%)	6 (50%)	2 (50%)	2 (17%)	11 (31%)
Hispanic/Latino N (%)	0	1 (25%)	1 (8.3%)	0	1 (8%)	3 (8%)
American Indian N (%)	0	0	0	1 (25%)	0	1 (3%)
Declined/NA	2 (50%)	2 (50%)	2 (17%)	0	0	6 (17%)
Ethnicity						
Non-Hispanic N (%)	2 (50%)	1 (25%)	7 (58%)	3 (75%)	11 (92%)	24 (67%)
Hispanic N (%)	2 (50%)	3 (75%)	5 (42%)	1 (25%)	1 (8%)	12 (33%)
ART Duration (months)						
Median (IQR)	146 (96-209)	94 (79-111)	132 (81-167)	173 (161-188)	162 (106-204)	144 (97-179)
Duration of virus suppression (months)						
Median (IQR)	76 (51-96)	62 (35-95)	79 (64-89)	77 (41-101)	70 (38-91)	75 (44-93)

*Percentage totals may not add up to 100 due to rounding. †IQR, interquartile range

Supplementary Table 2 – HEAL Participant Characteristics

Supplementary Table 2. HEAL Participant Characteristics

Characteristic	Cohort
Participants, N	11
Age	
Median (IQR†)	56 (55-62)
Sex	
Male (%)	7 (64%)
Race	
Black N (%)	6 (55%)
White N (%)	3 (27%)
Hispanic/Latino** N (%)	0
American Indian N (%)	0
Other	2 (18%)
Ethnicity	
Non-Hispanic N (%)	9 (82%)
Hispanic N (%)	2 (18%)
Duration of viral suppression (months)	
Median (IQR)	66 (26-83)

†IQR, interquartile range. ** Participants who reported Hispanic ethnicity identified their race as "Other."
